# Deproto-metallation of N-arylated pyrroles and indoles using a mixed lithium–zinc base and regioselectivity-computed CH acidity relationship

**DOI:** 10.3762/bjoc.11.160

**Published:** 2015-08-24

**Authors:** Mohamed Yacine Ameur Messaoud, Ghenia Bentabed-Ababsa, Madani Hedidi, Aïcha Derdour, Floris Chevallier, Yury S Halauko, Oleg A Ivashkevich, Vadim E Matulis, Laurent Picot, Valérie Thiéry, Thierry Roisnel, Vincent Dorcet, Florence Mongin

**Affiliations:** 1 Equipe Chimie et Photonique Moléculaires, Institut des Sciences Chimiques de Rennes, UMR 6226, CNRS-Université de Rennes 1, Bâtiment 10A, Case 1003, Campus de Beaulieu, 35042 Rennes, France; 2 Laboratoire de Synthèse Organique Appliquée, Faculté des Sciences, Université d’Oran 1 Ahmed Ben Bella, BP 1524 El M’Naouer, 31000 Oran, Algeria; 3 UNESCO Chair of Belarusian State University, 14 Leningradskaya Str., Minsk, 220030, Belarus; 4 Research Institute for Physico-Chemical Problems of Belarusian State University, 14 Leningradskaya Str., Minsk, 220030, Belarus; 5 Laboratoire Littoral Environnement et Sociétés, UMRi CNRS 7266, Université de La Rochelle, 17042 La Rochelle, France,; 6 Centre de Diffractométrie X, Institut des Sciences Chimiques de Rennes, UMR 6226, CNRS-Université de Rennes 1, Bâtiment 10B, Campus de Beaulieu, 35042 Rennes, France

**Keywords:** CH acidity, indoles, iodolysis, mixed lithium–zinc bases, pyrroles

## Abstract

The synthesis of N-arylated pyrroles and indoles is documented, as well as their functionalization by deprotonative metallation using the base in situ prepared from LiTMP and ZnCl_2_·TMEDA (1/3 equiv). With *N*-phenylpyrrole and *-*indole, the reactions were carried out in hexane containing TMEDA which regioselectively afforded the 2-iodo derivatives after subsequent iodolysis. With pyrroles and indoles bearing N-substituents such as 2-thienyl, 3-pyridyl, 4-methoxyphenyl and 4-bromophenyl, the reactions all took place on the substituent, at the position either adjacent to the heteroatom (S, N) or *ortho* to the heteroatom-containing substituent (OMe, Br). The CH acidities of the substrates were determined in THF solution using the DFT B3LYP method in order to rationalize the experimental results.

## Introduction

Pyrrole occurs in very important natural products such as tetrapyrrolic (linear) bilirubinoids, and (cyclic) porphyrins and corrins, as well as in pharmaceuticals (e.g., pyrrolnitrin, zomepirac) and polymers (e.g., photovoltaic cells). Indole is similarly present in numerous natural products (e.g., tryptophan, melanin, bufotenin, psilocin, indican) including bioactive products (e.g., strychnine, brucine, yohimbine, reserpine, vincamine, ergotamine, lysergic acid), as well as in pharmaceuticals (e.g., indomethacin, iprindole), agrochemicals (e.g., auxins, pyroquilon), and dyes and pigments (e.g., indigo, indocyanines) [1–2].

The deprotonative metallation [3–7] is a valuable tool for the regioselective functionalization of aromatic heterocycles such as pyrroles [8] and indoles [9]. A few examples concern the reaction of *N*-arylpyrroles and -indoles.

Studies show that, depending on the reaction conditions, two protons of *N*-phenylpyrrole (at the 2 and 2’ position) can be abstracted by a base. Kinetic conditions employing butyllithium activated by *N*,*N*,*N’*,*N’*-tetramethylethylenediamine (TMEDA) in diethyl ether lead to the 2,2’-dilithiated product [10–11]. In contrast, monolithiation at the 2 position is noted by using (i) the same base in diethyl ether at room temperature and long reaction times or in refluxing hexane (thermodynamic conditions) [11–12] or (ii) with butyllithium activated by potassium *tert*-butoxide (LICKOR) in tetrahydrofuran (THF) at −75 °C [11,13]. *N*-Arylpyrroles substituted on their six-membered ring by methoxy [14], halogen [15–16], alkyl [17–18], or trifluoromethyl [19–20] groups have been the topic of more recent studies. The reactions are in general performed at low temperatures (between −75 and 0 °C) and do not tolerate the presence of reactive functional groups.

Mono- and dilithiation of *N*-phenylindole takes place by using TMEDA-activated butyllithium, respectively in toluene at 100 °C (1 equiv of base) [21] and in diethyl ether at −70 °C (2 equiv) [22].

In the course of the last fifteen years, combinations of lithium reagents and softer metal compounds have established themselves as appropriate tools to deproto-metallate sensitive aromatic compounds [23–30]. In the search of bases usable at room temperature, we developed pairs of metal amides able to behave synergically in such reactions. In particular, the lithium–zinc basic mixture obtained in situ by mixing ZnCl_2_·TMEDA and LiTMP (TMP = 2,2,6,6-tetramethylpiperidino) in a 1 to 3 ratio [31], and for which the 1:1 LiTMP·2LiCl(±TMEDA)–Zn(TMP)_2_ composition was given [32], proved to be a ‘superbase’. Indeed, its reactivity is higher than that of the separate LiTMP and Zn(TMP)_2_) when used to functionalize sensitive aromatic compounds such as heterocycles [31,33–41].

**Figure 1:**
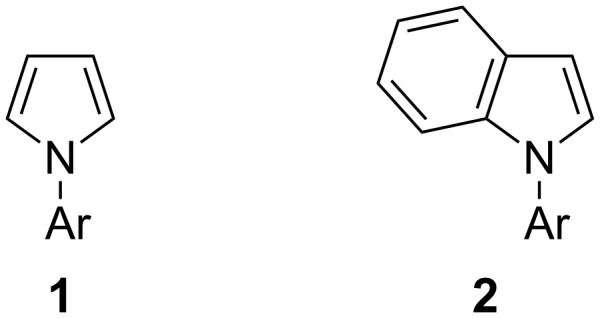
Substrates involved in deproto-metallation reaction.

Herein, we report our efforts to functionalize N-arylated pyrroles and indoles through deproto-metallation using this mixed lithium–zinc base (Figure 1). We showed earlier, for related substrates, the impact of the different hydrogen acidities on the regioselectivity of the reaction [37–38,40,42–43]. Based on these results, we here use CH acidities of the aromatic substrates in THF (calculated using the homodesmic reaction approach within the density functional theory (DFT) framework) to attempt a rationalization of the practical results.

## Results

### Synthetic aspects

**Figure 2:**
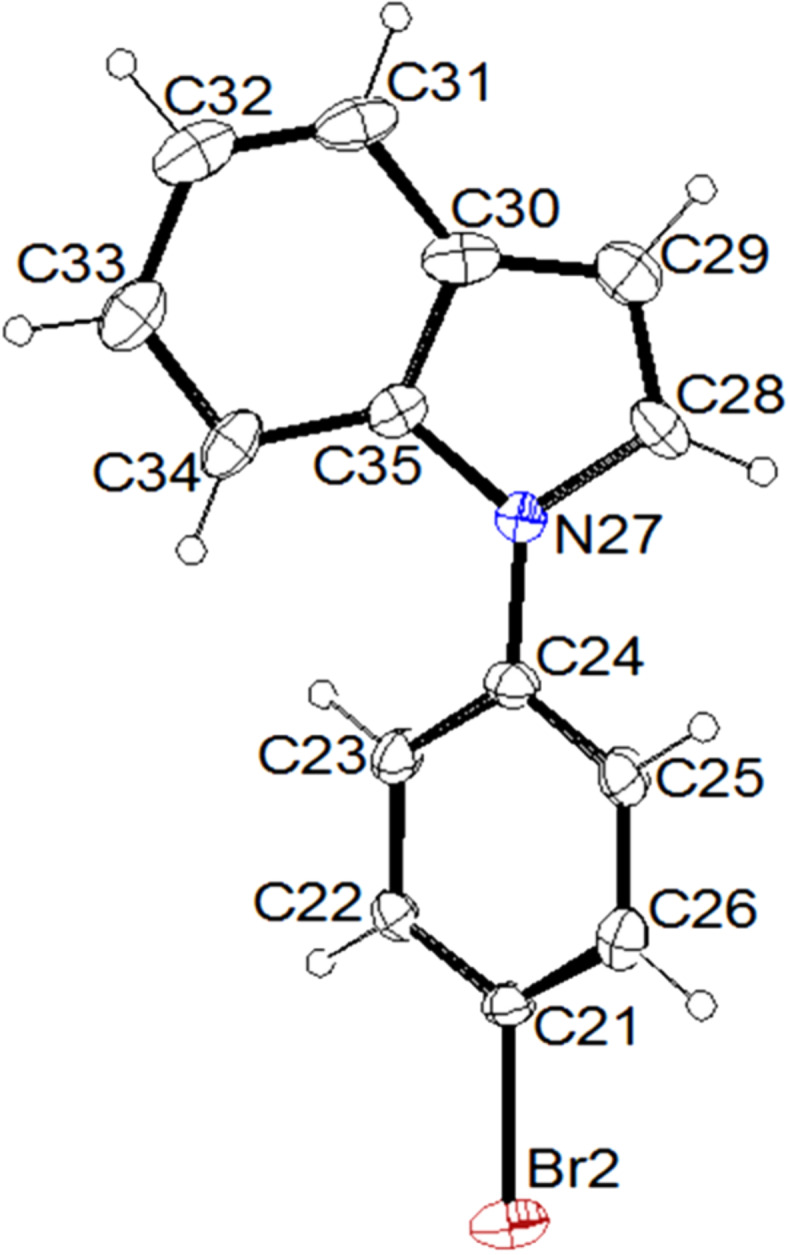
ORTEP diagram (30% probability) of **2e**.

**Scheme 1:**
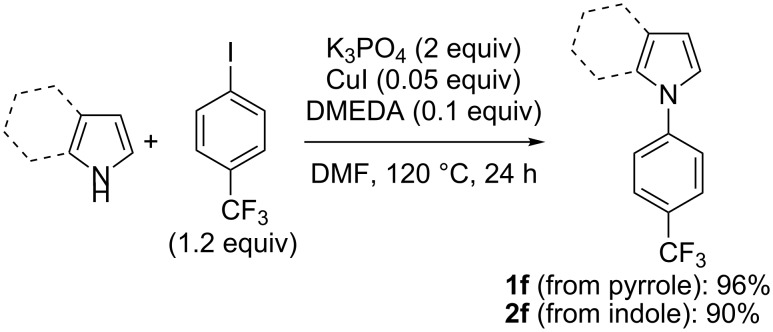
Synthesis of the azole substrates **1f** and **2f**.

In order to prepare the different pyrrole and indole substrates **1** and **2**, the unsubstituted azoles were reacted with aryl and heteroaryl iodides under copper catalysis. As far as the derivatives **1a**,**b**, **1d**,**e** and **2a–e** are concerned, the reactions were carried out by using 1.5 equiv of the azole, 0.2 equiv of copper, 2 equiv of caesium carbonate as the base, in acetonitrile at reflux [44] (Table 1, Figure 2, Supporting Information File 1). Varying yields were obtained, with aryl iodides substituted by electron-withdrawing groups in general favouring the reaction. In contrast, much lower yields were noted when using aryl bromides. For the synthesis of derivatives **1f** and **2f**, we rather employed 1.2 equiv of the aryl halide, 0.05 equiv of copper(I) iodide, 0.10 equiv of *N*,*N’*-dimethylethylenediamine (DMEDA), 2 equiv of tripotassium phosphate in dimethylformamide as the solvent at 120 °C [45]. Under these conditions, the *N*-(4-(trifluoromethyl)phenyl)-substituted azoles were isolated in high yields (Scheme 1).

**Table 1:** Synthesis of the azole substrates **1a**,**b**, **1d**,**e** and **2a–e**.

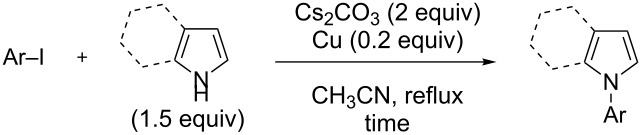				

Entry	Ar	Azole	Time	Product, yield (%)^a^

1	2-thienyl	pyrrole	16 h	**1a**, 75 (20)^b^
2	3-pyridyl	pyrrole	32 h	**1b**, 65 (26)^c^
3	4-MeOC_6_H_4_	pyrrole	72 h	**1d**, 46
4	4-BrC_6_H_4_	pyrrole	72 h	**1e**, 81
5	2-thienyl	indole	48 h	**2a**, 50
6	3-pyridyl	indole	48 h	**2b**, 70
7	Ph	indole	24 h	**2c**, 30
8	4-MeOC_6_H_4_	indole	72 h	**2d**, 15
9	4-BrC_6_H_4_	indole	56 h	**2e**, 60

^a^After purification. ^b^Using 2-bromothiophene. ^c^Using 3-bromopyridine.

**Scheme 2:**
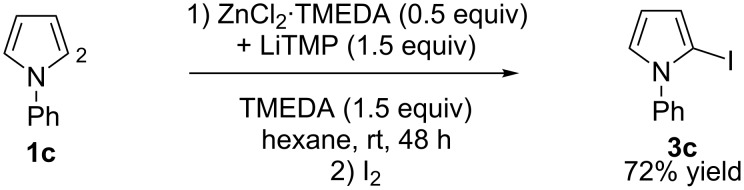
Deproto-metallation of **1c** followed by iodolysis [33].

In a previous study [33], we have shown that hexane containing *N*,*N*,*N’*,*N’*-tetramethylethylenediamine (TMEDA) is a suitable solvent to perform the C2 deproto-metallation with the TMP-based lithium–zinc as the base of commercially available *N*-phenylpyrrole (**1c**). The result has been evidenced by subsequent iodolysis (Scheme 2). Nevertheless, for numerous substrates, THF is an alternative solvent that allows the reactions to be finished after 2 h contact at room temperature [33–34,36].

Among the different methods of trapping that can be employed after deproto-zincation (interception with aldehydes, phenyl disulfide and allyl bromide, or palladium-catalyzed cross-coupling with aryl halides) [35], we chose the iodolysis, which is the most efficient quench. In addition, it offers the possibility of a subsequent functionalization of the formed aryl iodides by recourse to transition metal-catalyzed coupling reactions [37].

**Scheme 3:**
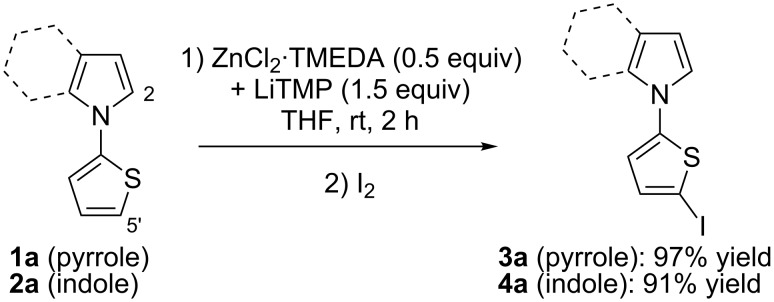
Deproto-metallation of **1a** and **2a** followed by iodolysis.

When bis-heterocyclic compound **1a** was reacted in THF for 2 h at room temperature with the lithium–zinc base, in situ prepared from ZnCl_2_·TMEDA (0.5 equiv) and LiTMP (1.5 equiv), a selective deprotonation at the thiophene ring took place. After subsequent interception with iodine, *N*-(5-iodo-2-thienyl)pyrrole (**3a**) was isolated in nearly quantitative yield. Under the same reaction conditions, the corresponding indole substrate **2a** was similarly converted into *N*-(5-iodo-2-thienyl)indole (**4a**) (Scheme 3).

**Scheme 4:**
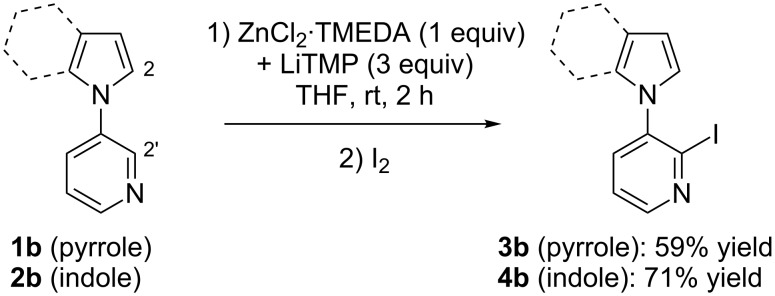
Deproto-metallation of **1b** and **2b** followed by iodolysis.

Starting from the bis-heterocycles **1b** and **2b**, better results were obtained by doubling the amount of base (using 1 equiv of ZnCl_2_·TMEDA and 3 equiv of LiTMP). Under the reaction conditions employed above, the deprotonation occurred on the pyridine ring, at the 2 position adjacent to the azole substituent. The corresponding monoiodides **3b** and **4b** were isolated in 59 and 71% yield, respectively along with unreacted starting material in both cases (Scheme 4).

**Scheme 5:**
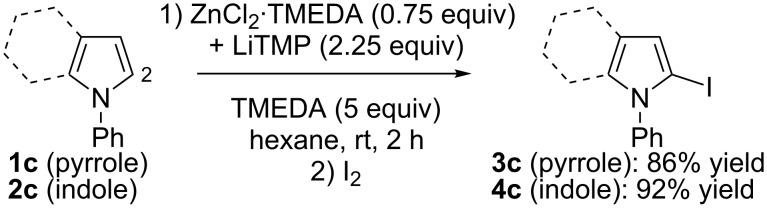
Deproto-metallation of **1c** and **2c** followed by iodolysis.

**Figure 3:**
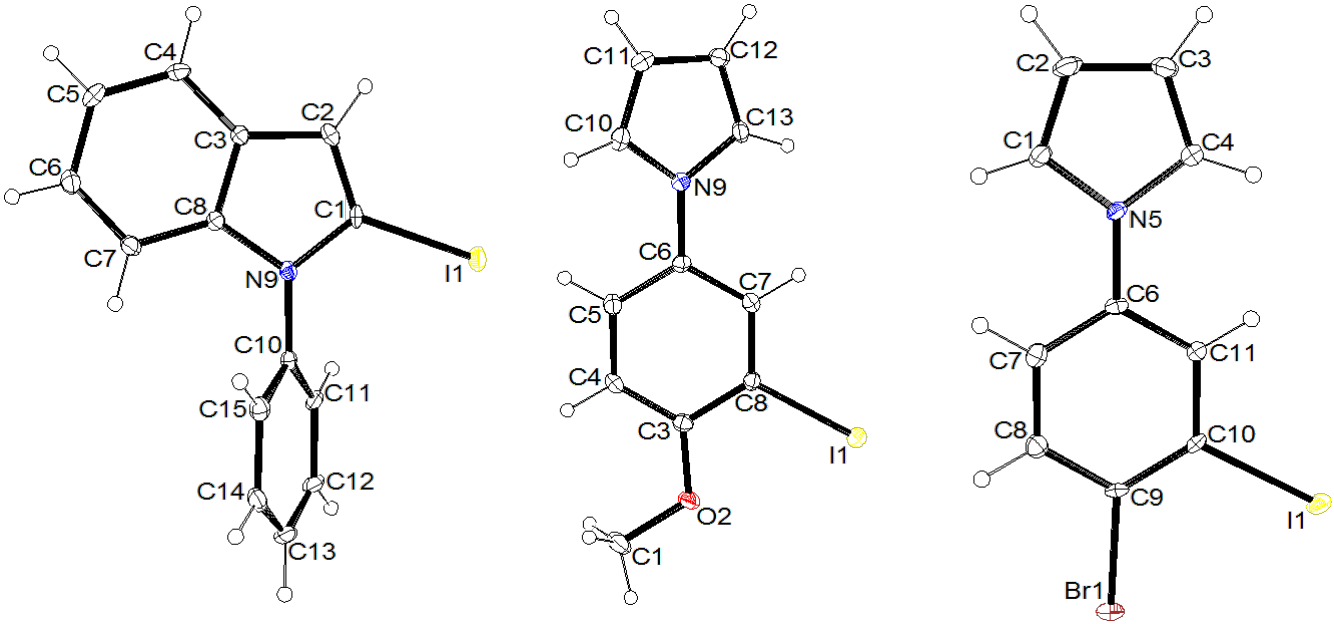
ORTEP diagrams (30% probability) of **4c**, **3d** and **3e**.

As previously observed for *N*-phenylpyrrole (**1c**) [33], the best solvent for the deprotonation of *N*-phenylindole (**2c**) is hexane containing TMEDA (5 equiv). Optimization of the base amount led to employ ZnCl_2_·TMEDA (0.75 equiv) and LiTMP (2.25 equiv). After 2 h contact at room temperature and subsequent iodolysis, the *N*-phenylated 2-iodopyrrole **3c** and 2-iodoindole **4c** were produced in 86 and 92% yield, respectively (Scheme 5). The structure of **4c** was unambiguously identified by X-ray diffraction (Figure 3, left, see Supporting Information File 1).

**Scheme 6:**
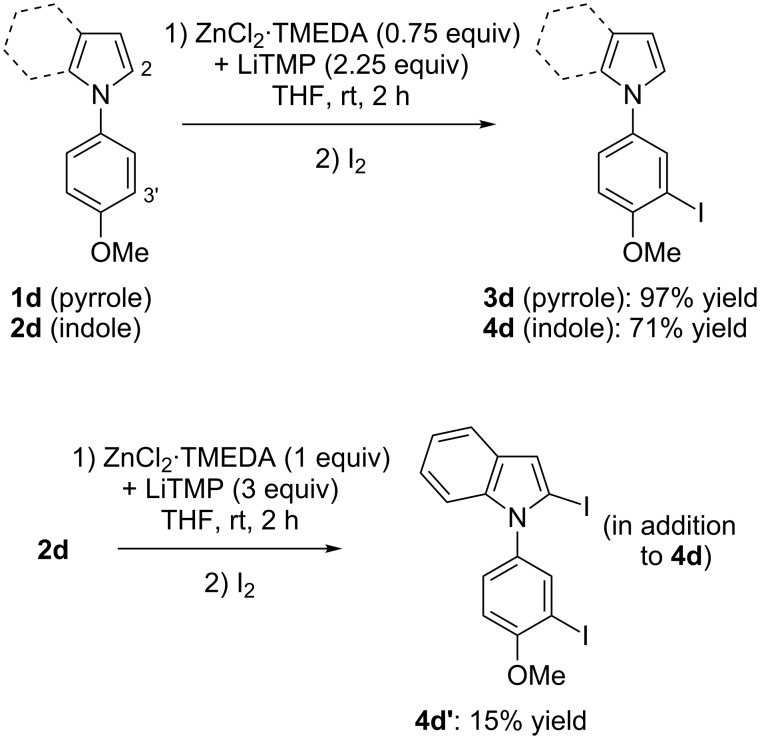
Deproto-metallation of **1d** and **2d** followed by iodolysis.

It is known that anisole can be *ortho*-deprotonated using the lithium–zinc base in THF [35]. It was thus of interest to involve *N*-(4-methoxyphenyl)-substituted azole substrates in the deprotonation–iodination sequence. Under similar conditions, deprotonation of **1d** and **2d** takes place next to the methoxy group, affording the monoiodides **3d** and **4d** in satisfying yields after iodolysis. Increasing the amount of base to 1 equiv of zinc in the case of **2d** (in order to reduce the amount of recovered starting material at the end of the reaction) led to the isolation of the diiodide **4d’**, resulting from a two-fold deprotonation at both the 2 and 3’ position (the rest being the monoiodide **4d**) (Scheme 6). The iodide **3d** was identified by X-ray diffraction from suitable crystals (Figure 3, middle, see Supporting Information File 1).

**Scheme 7:**
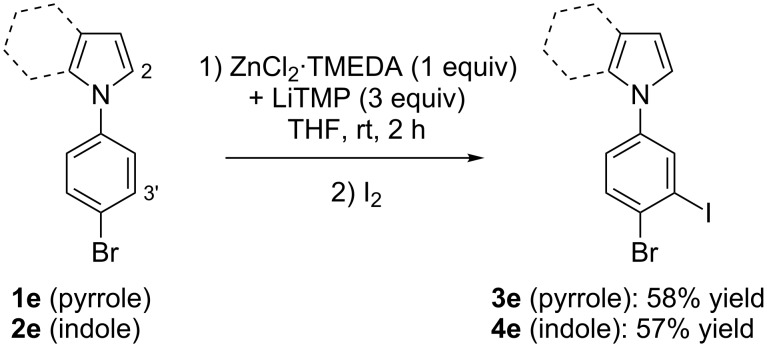
Deproto-metallation of **1e** and **2e** followed by iodolysis.

Whereas various attempts to deprotonate bromobenzene using the lithium–zinc base failed, a result probably due to degradation of the deproto-metallated compound through benzyne formation [46], it proved possible to accumulate at room temperature the arylmetal compounds formed from the *N*-(4-bromophenyl)azoles **1e** and **2e**, as demonstrated by trapping with iodine. The best results (minimum of degradation) were observed by using ZnCl_2_·TMEDA (1 equiv) and LiTMP (3 equiv). Under these conditions the iodides **3e** and **4e** were obtained in 58 and 57% yield, respectively (Scheme 7). The structure of product **3e** was unequivocally identified by X-ray diffraction (Figure 3, right, see Supporting Information File 1).

The reactions starting from **1a**–**e** and **2a**–**e** led to predominant products. In contrast, in the case of **1f** and **2f**, complex mixtures containing two monoiodides and one diiodide (unidentified regioselectivity) were obtained together with recovered starting material.

**Scheme 8:**
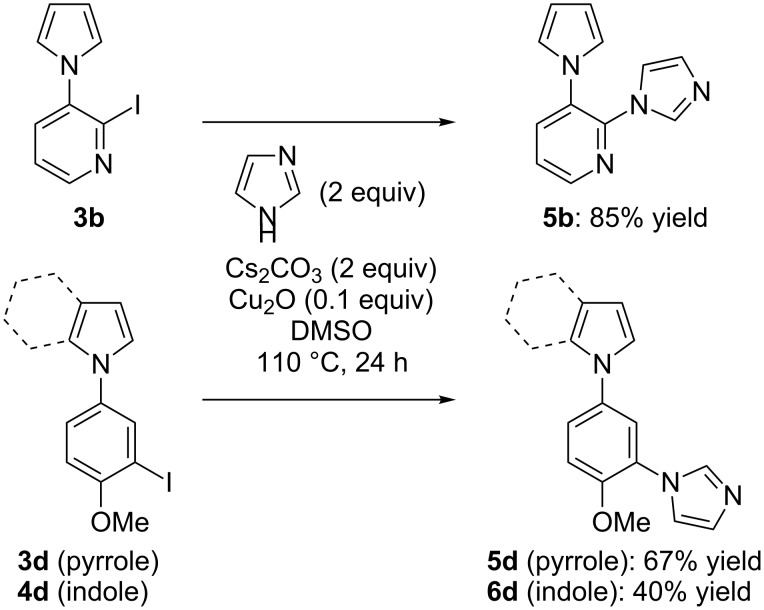
N-arylation of the iodides **3b**, **3d** and **4d**.

**Figure 4:**
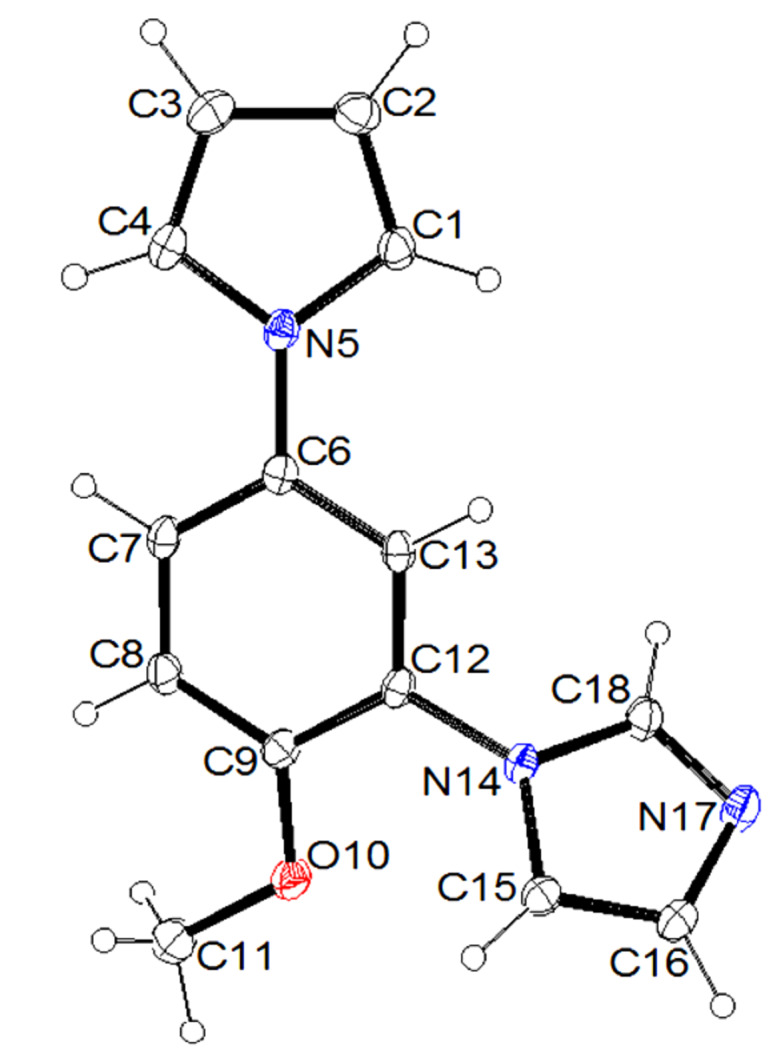
ORTEP diagram (30% probability) of **5d**.

In order to test the functionalization of the above synthesized aryl iodides through a subsequent transition-metal-catalyzed coupling reaction we subjected three of them to a copper-catalyzed N-arylation reaction. Thus, the iodides **3b**, **3d** and **4d** were reacted with 2 equiv of imidazole in the presence of 0.1 equiv of copper(I) oxide, 2 equiv of caesium carbonate as the base in dimethylsulfoxide (DMSO) at 110 °C [47]. Under these conditions, the imidazole-substituted compounds **5b**, **5d** and **6d** were generated in 85, 67 and 40% yield, respectively (Scheme 8, Figure 4, see Supporting Information File 1).

### Computational aspects

**Figure 5:**
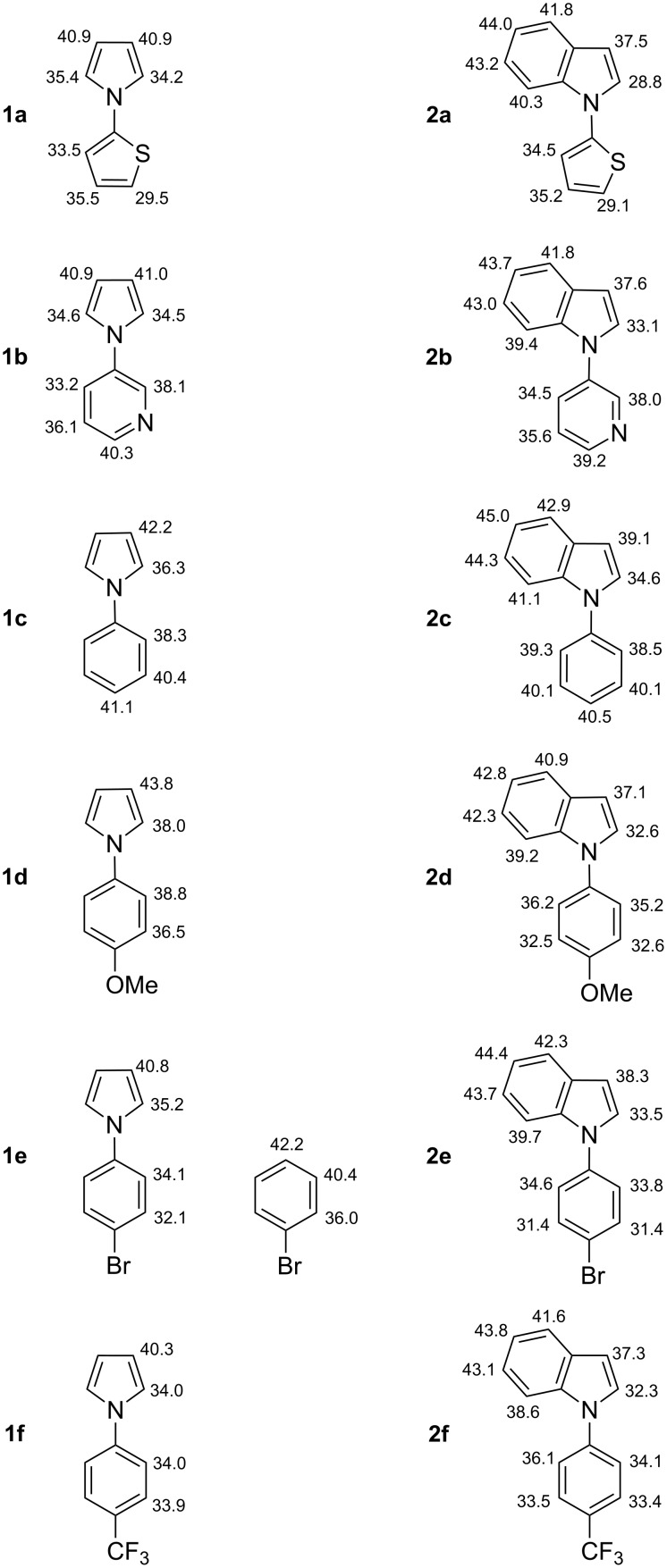
Calculated values of p*K*_a_(THF) of the compounds **1** and **2**, and bromobenzene.

There is a lack of data on the acidity of N-substituted pyrroles and indoles in the literature. NH acidity of unsubstituted pyrrole and indole in DMSO was measured by Bordwell [48] and computed by means of semi-empirical AM1 [49] and ab initio methods [50]. The p*K*_a_ values for *N*-methylindole, *N*-methylpyrrole and *N*-(dimethylamino)pyrrole as CH acids in THF were experimentally determined by Fraser et al [51]. The latter are more related to our goals and were found to be in good agreement with those computed within the DFT framework recently [42,52]. The results of quantum chemical calculations on CH acidity of the different N-arylated pyrroles **1** and indoles **2**, obtained both for THF solution (Figure 5) and gas phase (see Supporting Information File 2), are presented in the current paper.

A potential acidity of the methoxy groups in substrates **1d** and **2d** was ignored here since there was no sign of their deprotonation under the experimental conditions and as a consequence their acidity was expected to be substantially lower. The data in Figure 5 refer to the most stable rotamer of the compounds. Thus, potential energy surface (PES) scans allowed us to state that both **2a** and **2b** are likely to adopt a form in which the heteroatoms are far away from the benzo-fragment.

The calculated values of gas-phase acidity of the investigated compounds (see Supporting Information File 2) lie within the range of 364.4–394.3 kcal mol^−1^ which is typical for weak CH acidic compounds while the p*K*_a_ values in THF solution (Figure 5) covered a 28.8–45.0 span. These data allow the assignment of potential deprotonation sites in the investigated substrates. Also the correlation between gas-phase Δ*G*_acid_ and the p*K*_a_(THF) values can be tracked. In all cases, the CH acidity increases when changing from electron-donating groups to more electron-withdrawing ones. One can easily see that for both pyrroles and indoles the most acidic hydrogen is at the 2 position of the azole part, while for an aryl substituent it depends on its nature.

## Discussion

The results observed in the course of these deproto-metallation reactions complete previously reported studies using alkali metal bases with similar substrates [36–41]. The calculations of the CH acidities in THF (Figure 5) allow us to comment on the regioselectivities observed in the different studies.

To our knowledge, the *N*-(2-thienyl) azoles **1a** and **2a** (Scheme 3 and Figure 5) have never been subjected to deproto-metallation before. If we only consider the reaction of **1a**, it is conceivable that the regioselectivity only results from an attack of the lithium–zinc base at the most acidic site. In contrast, the regioselectivity observed in case of substrate **2a** cannot be explained by the p*K*_a_ values as the only reason. Otherwise, deprotonation would have been also observed at the 2 position of the indole ring. Whereas the azole nitrogen in **2a** cannot coordinate a metal through its electron lone pair (it is delocalized within the aromatic π-electron ring system), a coordination by the sulfur atom of the thiophene substituent could take place with a possible decrease of neighbouring p*K*_a_ values.

As for **1a** and **2a**, deproto-metallation has not yet been reported for **1b** and **2b** (Scheme 4 and Figure 5). In the case of these substrates, it is clear that deprotonation takes place after coordination of the pyridine nitrogen to a metal; otherwise, the reactions would have been observed at the 2 position of the azole ring. Among both positions adjacent to the pyridine nitrogen, the most acidic is attacked.

*N*-Phenylpyrrole (**1c**, Scheme 5 and Figure 5) does not have any atom capable of coordinating a metal. Thus, under thermodynamic conditions (using TMEDA-activated butyllithium either in diethyl ether at room temperature after long reaction times or in refluxing hexane [11–12]; using LICKOR in THF at −75 °C [11,13]), the most acidic site next to the azole nitrogen is affected. Using TMEDA-activated butyllithium in diethyl ether under kinetic conditions leads to 2,2’-dilithiation [10–11]. In this case, the intermediate 2-lithiated compound can be involved in aggregates favouring deprotonation at a neighbouring site to afford the 2,2’-dilithiated derivative. The 2,2’-dimetallated derivative of **1c** has also been noted using the lithium–zinc base, for example when the reaction is performed in THF (favoured at reflux temperature) [33]. In this case, the dimetallated compound does not correspond to a kinetic but to a thermodynamic compound. This could be due to the formation of a stable zinc metallacycle, as suggested in an earlier paper [37]. As previously reported [33], dideprotonation can be suppressed by using hexane containing TMEDA instead of THF and both compounds **1c** and **2c** are functionalized at their most acidic position (Scheme 5 and Figure 5).

The deprotolithiation of *N*-(4-methoxyphenyl)pyrrole (**1d**, Scheme 6 and Figure 5) by using chelates of butyllithium in THF at −75 °C for 1 h was documented by Faigl and co-workers in 1997 [14]. Whereas *N*,*N*,*N’*,*N”*,*N”*-pentamethyldiethylenetriamine (PMDETA) employed as ligand leads to reaction at the (maybe less hindered) 2 position (46% yield after conversion to the corresponding carboxylic acid), the most acidic position 3’ is attacked in the presence of TMEDA (18% yield after similar conversion) [14]. Using the lithium–zinc base also gives the 3’-metallated derivative, but more efficiently. *N*-(4-Methoxyphenyl)indole (**2d**, Scheme 6 and Figure 5) has two similarly acidified sites at C2 and C3’. The regioselective reaction at C3’ could result from a coordination of the methoxy group to a metal prior to deprotonation. Nevertheless, when the amount of base is increased, competitive dideprotonation takes place to furnish diiodides as previously noted with other azoles [37–38].

Compared with a methoxy group, the bromo substituent exhibits a stronger acidifying effect. By itself, this effect is not sufficient to enable the accumulation of a 2-metallated bromobenzene. However, when this *ortho*-bromine effect is combined with the *meta*-effect of a pyrrolyl group, the corresponding CH acidities are increased (see Figure 5), and things become different. Thus, whereas the direct deproto-lithiation of bromobenzene leads to highly unstable 2-bromophenyllithium, that of *N*-(4-bromophenyl)pyrrole (**1e**, Scheme 7 and Figure 5) is possible. By using LiTMP in THF at −75 °C for 1 h, Faigl and co-workers evidenced deprotonation next to the bromo group (21% yield after conversion to the corresponding carboxylic acid), i.e. at the most acidic position [16]. With the lithium–zinc base, the reactions from the *N*-(4-bromophenyl) azoles **1e** and **2e** (Scheme 7 and Figure 5) occurred with the same regioselectivity, and proved possible with acceptable yields at room temperature.

The deprotolithiation of *N*-(4-(trifluoromethyl)phenyl)pyrrole (**1f**, Figure 5) was reported by Faigl and co-workers in 1999 [19]. By employing TMEDA-activated butyllithium in THF at −75 °C, the authors mainly noted proton abstraction next to the trifluoromethyl group. A competitive minor reaction at the 2 position of the pyrrole group can be rationalized by similar p*K*_a_ values at C2 and C3’ [19]. Indeed, when compared with a bromo substituent, the trifluoromethyl group similarly (and maybe more strongly) exhibits a long range acidifying effect, but less acidifies the *ortho* positions [53–54]. The lack of regioselectivity observed in the course of the reaction between the lithium–zinc base and the *N*-(4-(trifluoromethyl)phenyl)azole **1f** or **2f** (Figure 5) could be in relation with a reaction temperature (too high) not suitable to discriminate between similarly acidified positions.

### Antiproliferative activity in A2058 melanoma cells

**Figure 6:**
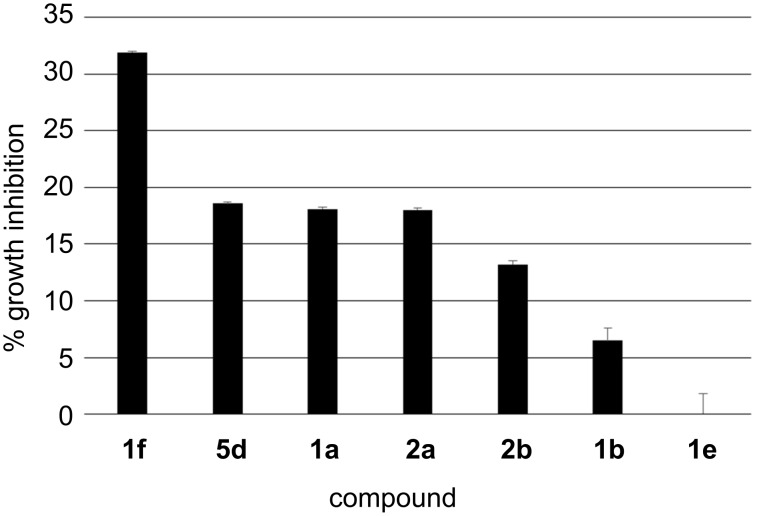
Antiproliferative activity (growth inhibition) of the tested compounds **1a**,**b**,**e**,**f**, **2a**,**b** and **5d** at concentration 10^−5^ M and 72 h in A2058 human melanoma cells.

The N-arylated pyrroles and indoles exerted low to moderate antiproliferative activity in A2058 melanoma cells (Figure 6). The best result was obtained with **1f** at 10^−5^ M, which induced 31.9 ± 0.1% growth inhibition in cells treated for 72 h. The linear N-arylated pyrroles **1b** and **1e** were poorly active. Arylation by a thiophene moiety (**1a** and **2a**) moderately increased the antiproliferative activity, with no significant difference between the pyrrole and indole derivatives.

## Conclusion

Unlike other azoles such as pyrazole and triazoles [37–38,40], pyrrole and indole do not possess any atom capable of coordinating metals. As a consequence, the corresponding CH acidities in THF solution, which were calculated using a continuum solvation model, better help in rationalizing the outcome of the deproto-metallation reactions.

**Figure 7:**
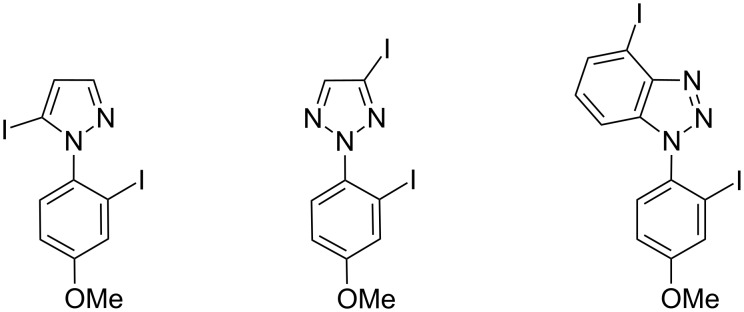
Iodides previously formed as major products from the corresponding *N*-(4-methoxyphenyl)azoles using the lithium–zinc base.

In addition, whereas *N*-(4-substituted phenyl)pyrazoles and -triazoles (e.g., with methoxy as substituent, Figure 7) can be deprotonated at C2’ for the same reason [37–38,40], the corresponding *N*-(4-substituted phenyl)pyrroles and -indoles are rather functionalized at C3’, next to the substituent.

## Experimental

**General methods.** All the reactions were performed under argon atmosphere. THF was distilled over sodium/benzophenone. ZnCl_2_·TMEDA was prepared as described previously [35]. Column chromatography was performed on silica gel (40–63 μm). Melting points were measured on a Kofler apparatus. IR spectra were taken on a Perkin-Elmer Spectrum 100 spectrometer. ^1^H and ^13^C nuclear magnetic resonance (NMR) spectra were recorded on a Bruker Avance III spectrometer at 300 and 75 MHz, respectively. ^1^H chemical shifts (δ) are given in ppm relative to the solvent residual peak, ^13^C chemical shifts are relative to the central peak of the solvent signal [55]. Mass spectra measurements were performed using a HP 6890 instrument.

**Crystallography.** The single crystals were studied with graphite monochromatized MoKα radiation (λ = 0.71073 Å). X-ray diffraction data were collected at *T* = 150(2) K using an APEXII Bruker-AXS diffractometer. The structure was solved by direct methods using the SIR97 program [56], and then refined with full-matrix least-square methods based on *F*^2^ (SHELX-97) [57] with the aid of the WINGX program [58]. All non-hydrogen atoms were refined with anisotropic atomic displacement parameters. Hydrogen atoms were finally included in their calculated positions. Molecular diagrams were generated by ORTEP-3 (version 2.02) [59].

**Procedure 1 for the synthesis of the N-arylated pyrroles and indoles 1a,b,d,e and 2a–e** [44]*.* To azole (6.0 mmol) and aryl halide (4.0 mmol) in acetonitrile (20 mL) were successively added Cu (50 mg, 0.80 mmol), Cs_2_CO_3_ (2.6 g, 8.0 mmol) and, in the case of aryl bromides, KI (99 mg, 6.0 mmol). The mixture was stirred under argon at acetonitrile reflux temperature (the reaction time is given in the product description) before dilution with AcOEt (40 mL) and filtration. Concentration under reduced pressure and purification by chromatography on silica gel (the eluent is given in the product description) led to the expected compounds.

**Procedure 2 for the synthesis of the N-arylated pyrroles and indoles 1f and 2f** [45]*.* To azole (10 mmol) and aryl halide (12 mmol) in DMF (5 mL) were successively added CuI (95 mg, 0.50 mmol), K_3_PO_4_ (4.2 g, 20 mmol) and DMEDA (0.11 mL, 1.0 mmol). The mixture was stirred under argon at 120 °C (the reaction time is given in the product description) before dilution with AcOEt (40 mL) and filtration. Concentration under reduced pressure and purification by chromatography on silica gel (the eluent is given in the product description) led to the compounds described below.

**General procedure 3 for the deprotonative metallation followed by iodination** (analogous to that described in [60]). To a stirred, cooled (0 °C) solution of 2,2,6,6-tetramethylpiperidine (0.25 mL, 1.5 mmol) in THF (2–3 mL) were successively added BuLi (about 1.6 M hexanes solution, 1.5 mmol) and, 5 min later, ZnCl_2_·TMEDA (0.13 g, 0.50 mmol). The mixture was stirred for 15 min at 0 °C before introduction of the substrate (1.0 mmol) at 0–10 °C. After 2 h at room temperature, a solution of I_2_ (0.38 g, 1.5 mmol) in THF (4 mL) was added. The mixture was stirred overnight before addition of an aqueous saturated solution of Na_2_S_2_O_3_ (4 mL) and extraction with AcOEt (3 × 20 mL). The combined organic layers were dried over MgSO_4_, filtered and concentrated under reduced pressure. Purification by chromatography on silica gel (the eluent is given in the product description) led to the compounds **3a** and **4a**.

**General procedure 4 for the deprotonative metallation followed by iodination** (analogous to that described in [60]). To a stirred, cooled (0 °C) solution of 2,2,6,6-tetramethylpiperidine (0.25 mL, 1.5 mmol) in THF (2-3 mL) were successively added BuLi (about 1.6 M hexanes solution, 1.5 mmol) and, 5 min later, ZnCl_2_·TMEDA (0.13 g, 0.50 mmol). The mixture was stirred for 15 min at 0 °C before introduction of the substrate (0.5 mmol) at 0–10 °C. After 2 h at room temperature, a solution of I_2_ (0.38 g, 1.5 mmol) in THF (4 mL) was added. The mixture was stirred overnight before addition of an aqueous saturated solution of Na_2_S_2_O_3_ (4 mL) and extraction with AcOEt (3 × 20 mL). The combined organic layers were dried over MgSO_4_, filtered and concentrated under reduced pressure. Purification by chromatography on silica gel (the eluent is given in the product description) led to the compounds **3b**,**e** and **4b**,**d'**,**e**.

**General procedure 5 for the deprotonative metallation followed by iodination** (analogous to that described in [60]). To a stirred, cooled (0 °C) solution of 2,2,6,6-tetramethylpiperidine (0.25 mL, 1.5 mmol) in hexane (2–3 mL) containing TMEDA (0.50 mL, 3.3 mmol) were successively added BuLi (about 1.6 M hexanes solution, 1.5 mmol) and, 5 min later, ZnCl_2_·TMEDA (0.13 g, 0.50 mmol). The mixture was stirred for 15 min at 0 °C before introduction of the substrate (0.67 mmol) at 0–10 °C. After 2 h at room temperature, a solution of I_2_ (0.38 g, 1.5 mmol) in THF (4 mL) was added. The mixture was stirred overnight before addition of an aqueous saturated solution of Na_2_S_2_O_3_ (4 mL) and extraction with AcOEt (3 × 20 mL). The combined organic layers were dried over MgSO_4_, filtered and concentrated under reduced pressure. Purification by chromatography on silica gel (the eluent is given in the product description) led to the compounds **3c** and **4c**.

**General procedure 6 for the deprotonative metallation followed by iodination** (analogous to that described in [60]). To a stirred, cooled (0 °C) solution of 2,2,6,6-tetramethylpiperidine (0.25 mL, 1.5 mmol) in THF (2–3 mL) were successively added BuLi (about 1.6 M hexanes solution, 1.5 mmol) and, 5 min later, ZnCl_2_·TMEDA (0.13 g, 0.50 mmol). The mixture was stirred for 15 min at 0 °C before introduction of the substrate (0.67 mmol) at 0–10 °C. After 2 h at room temperature, a solution of I_2_ (0.38 g, 1.5 mmol) in THF (4 mL) was added. The mixture was stirred overnight before addition of an aqueous saturated solution of Na_2_S_2_O_3_ (4 mL) and extraction with AcOEt (3 × 20 mL). The combined organic layers were dried over MgSO_4_, filtered and concentrated under reduced pressure. Purification by chromatography on silica gel (the eluent is given in the product description) led to the compounds **3d** and **4d**.

**Procedure 7 for the N-arylation of imidazole** [47]*.* A mixture of the prepared iodide (1.0 mmol), Cu_2_O (0.10 g, 0.10 mmol), Cs_2_CO_3_ (0.65 g, 2.0 mmol), imidazole (0.14 g, 2.0 mmol) and DMSO (0.5 mL) was stirred for 24 h at 110 °C under argon. After cooling to room temperature, the mixture was diluted with AcOEt (10 mL) and filtered over celite^®^. Washing with AcOEt, removal of the solvent and purification by chromatography on silica gel (the eluent is given in the product description) led to the compounds **5b**,**d** and **6d**.

**Computational procedure.** The DFT calculations were performed using GAUSSIAN 03W package [61]. The B3LYP formalism was employed. All optimized geometries were obtained using the 6-31G(d) basis set without any symmetry constraints implied. Vibrational frequencies were calculated at the same level of theory in order to characterize stationary points and to calculate zero-point vibrational energies (ZPVE) and thermal corrections. The total energy of species was found using the 6-311+G(d,p) basis set and tight convergence criteria. Further, the gas-phase Gibbs energies (*G*^0^_298_) were calculated using the following equation:

*G*^0^_298_ = *E* + ZPVE + *H*^0^_0→298_ – *TS*^0^_298_

The gas-phase acidity Δ*G*_acid_ was defined as the Gibbs energy of deprotonation of the particular substrate R–H (R–H_(g)_ → R^−^_(g)_ + H^+^_(g)_):

Δ*G*_acid_ = *G*^0^_298_(R^−^) + *G*^0^_298_(H^+^) − *G*^0^_298_(R–H).

The solvent influence was simulated within the polarized continuum model (PCM) with the default parameters for THF. The cavity was built up using atomic radii from the UFF force field. The PCM energies *E*_PCM_ were calculated at the B3LYP/6-311+G(d,p) level using geometries optimized for isolated structures. The Gibbs energy in solution *G*_sol_ was calculated for each species by the formula:

*G*_sol_ = *G*^0^_298_ + *E*_PCM_ − *E*.

The following homodesmic reaction was composed for the p*K*_a_ values calculation:

R–H_(s)_ + Het^−^_(s)_ → R^−^_(s)_ + Het–H_(s)_,

where Het–H is *N*-methylindole. The latter was chosen as reference compound due to its structural similarity and since its p*K*_a_(THF) = 38.1 found by Fraser et al [51] was expected to be close to those for our substrates. Consequently, the Gibbs energy of the homodesmic reaction (Δ*G*_r,sol_) and the p*K*_a_ value are related by the following equation:


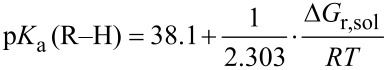


**Biological evaluation.** The antiproliferative activity of N-arylated pyrrole and indole derivatives was studied in the A2058 (ATCC^®^ CRL-11147) cell line as described previously [60]. A2058 cells are highly invasive human epithelial adherent melanoma cells, derived from lymph nodes metastatic cells obtained from a 43 year old male patient. They are tumorigenic at 100% frequency in nude mice, and considered as very resistant to anticancer drugs. All cell culture experiments were performed at 37 °C. Cells were grown to confluence in 75 cm² flasks in DMEM supplemented with 10% fetal calf serum (FCS) and 1% penicillin–streptomycin (Dominique Dutscher, France), in a 5% CO_2_ humidified atmosphere. Molecules were solubilized in DMSO at 10^−3^ M and diluted in the cell culture medium to obtain 2·10^−5^ M solutions. Confluent cells were trypsinized and centrifuged in FCS at 1500*g* for 5 min. The supernatant containing trypsin was discarded and the cell pellet was resuspended in cell culture medium to obtain a 4·10^4^ cell·mL^−1^ suspension. At *t*_0_, 50 µL of the 2·10^−5^ M test solutions were deposited in a 96-wells flat bottom microplate, and 50 µL of the cell suspension were added. The 2000 cells were then grown for 72 h in the cell culture medium containing 10^−5^ M molecules. At *t*_72_, 20 µL of a 5 g·L^−1^ MTT solution were added to each well of the microplate, allowing living cells containing a functional mitochondrial succinate dehydrogenase to metabolize MTT to the corresponding blue formazan salt for 4 h. The cell culture medium was removed using an Eppendorf epMotion 5070 pipeting robot (Eppendorf, France) and formazan crystals were dissolved in 200 µL DMSO. Microplates were placed at 37 °C for 5 min to solubilize formazan crystals and absorbance was read at 550 nm using a VERSAmax microplate reader (Molecular devices, France). The percentage of growth inhibition was calculated as GI (%) = 100 − ((A_550nm_ sample − A_550nm_ BG)/(A_550nm_ control − A_550nm_ BG))·100, with:

- A_550 nm_ sample as median absorbance of 8 wells containing cells treated with 10^−5^ M compound solution
- A_550 nm_ BG as median background absorbance of 8 wells containing control cell culture medium + 1% DMSO
- A_550 nm_ control as median absorbance of 8 wells containing cells grown in control cell culture medium + 1% DMSO.

The data are expressed as GI (%) + sem (%) from 3 independent assays.

## Supporting Information

File 1 Experimental description of the synthesized compounds, ^1^H and ^13^C NMR spectra, calculated values of the Gibbs energies Δ*G*_acid_ [kcal·mo1^−1^] for deprotonation, selected Cartesian coordinates of molecular geometry for the most stable rotamer forms optimized at B3LYP/6-31G(d) level of theory.

File 2 CIF files of **2e** (CCDC1402111), **4c** (1402112), **3d** (1402113), **3e** (1402114), and **5d** (1402115). These data can be obtained free of charge from The Cambridge Crystallographic Data Centre via http://www.ccdc.cam.ac.uk/data_request/cif.
