# Supplementary material for: Deproto-metallation of N-arylated pyrroles and indoles using a mixed lithium–zinc base and regioselectivity-computed CH acidity relationship
Source: Beilstein J Org Chem. 2015 Aug 24;11:1475–85. doi: 10.3762/bjoc.11.160 (PMC4578368; doi:10.3762/bjoc.11.160)
Supplement: File 1 — Experimental description of the synthesized compounds, 1H and 13C NMR spectra, calculated values of the Gibbs energies ΔGacid [kcal·mo1−1] for deprotonation, selected Cartesian coordinates of molecular geometry for the most stable rotamer forms optimized at B3LYP/6-31G(d) level of theory. [file Beilstein_J_Org_Chem-11-1475-s001.pdf]

Supporting Information

for

Deproto-metallation of N-arylated pyrroles and indoles using a mixed lithium–zinc base and regioselectivity-computed CH acidity relationship

Mohamed Yacine Ameur Messaoud[1,2], Ghenia Bentabed-Ababsa*[2], Madani Hedidi[1,2], Aïcha Derdour[2], Floris Chevallier[1], Yury S. Halauko*[3], Oleg A. Ivashkevich[3], Vadim E. Matulis[4], Laurent Picot*[5], Valérie Thiéry*[5], Thierry Roisnel[6], Vincent Dorcet[6] and Florence Mongin*[1]

Address: [1]Equipe Chimie et Photonique Moléculaires, Institut des Sciences Chimiques de Rennes, UMR 6226, CNRS-Université de Rennes 1, Bâtiment 10A, Case 1003, Campus de Beaulieu, 35042 Rennes, France, [2]Laboratoire de Synthèse Organique Appliquée, Faculté des Sciences, Université d'Oran 1 Ahmed Ben Bella, BP 1524 El M'Naouer, 31000 Oran, Algeria, [3]UNESCO Chair of Belarusian State University, 14 Leningradskaya Str., Minsk, 220030, Belarus, [4]Research Institute for Physico-Chemical Problems of Belarusian State University, 14 Leningradskaya Str., Minsk, 220030, Belarus, [5]Laboratoire Littoral Environnement et Sociétés, UMRi CNRS 7266, Université de La Rochelle, 17042 La Rochelle, France, and [6]Centre de Diffractométrie X, Institut des Sciences Chimiques de Rennes, UMR 6226, CNRS-Université de Rennes 1, Bâtiment 10B, Campus de Beaulieu, 35042 Rennes, France

Email: Ghenia Bentabed-Ababsa* - badri_sofi@yahoo.fr; Yury S. Halauko* - hys@tut.by; Laurent Picot* - laurent.picot@univ-lr.fr; Valérie Thiéry* - valerie.thiery@univ-lr.fr; Florence Mongin* - Florence.mongin@univ-rennes1.fr

*Corresponding author

Experimental description of the synthesized compounds, ^{1}H and ^{13}C NMR spectra, calculated values of the Gibbs energies ΔG_{acid} [kcal·mo1^{-1}] for deprotonation, selected Cartesian coordinates of molecular geometry for the most stable rotamer forms optimized at B3LYP/6-31G(d) level of theory

Table of Contents

Experimental descriptions of the synthesized compounds S3

^{1}H and ^{13}C NMR spectra of compounds **3a**, **4a**, **3b**, **4d'**, **3e**, **4e**, **4c**, **3d**,

4d, **5b**, **5d** and **6d** S10

Calculated values of the Gibbs energies $\Delta_{acid}G$ [kcal mol^{-1}] for deprotonation

at the corresponding positions of the investigated compounds S22

Cartesian coordinates of molecular geometries for most stable rotamer form

of selected compounds (on example of **1b** and **2b**, neutral molecule,

gas phase) optimized at B3LYP/6-31G(d) level of theory S23

***N*-(2-Thienyl)pyrrole (1a)** [1]**.** The procedure 1 (16 h reaction time) using pyrrole (0.42 mL) and 2-iodothiophene (0.44 mL) (or 2-bromothiophene (0.39 mL)) gave **1a** (eluent: heptane-CH_2Cl_2 95:5) in 75% (20%) yield as a beige powder: mp 57 °C; ^{1}H NMR ($CDCl_3$) δ 6.33 (t, 2H, *J* = 2.1 Hz), 6.91 (dd, 1H *J* = 3.6 and 1.5 Hz), 6.93-6.97 (m, 1H), 6.99 (d, 1H, *J* = 1.5 Hz), 7.01 (t, 2H, *J* = 2.1 Hz); ^{13}C NMR ($CDCl_3$) δ 110.5 (2CH), 115.6 (CH), 119.2 (2CH), 121.4 (CH), 126.2 (CH), 144.3 (C).

***N*-(3-Pyridyl)pyrrole (1b)** [2]**.** The procedure 1 (32 h reaction time) using pyrrole (0.42 mL) and 3-iodopyridine (0.82 g) (or 3-bromopyridine (0.39 mL)) gave **1b** (eluent: heptane-AcOEt 80:20) in 65% (26%) yield as a yellow oil: ^{1}H NMR ($CDCl_3$) δ 6.38 (t, 2H, *J* = 2.1 Hz), 7.07 (t, 2H, *J* = 2.3 Hz), 7.31 (dd, 1H *J* = 8.1 and 4.8 Hz), 7.64 (ddd, 1H, *J* = 8.4, 2.7 and 1.5 Hz), 8.47 (d, 1H, *J* = 4.5 Hz), 8.72 (d, 1H, *J* = 2.1 Hz); ^{13}C NMR ($CDCl_3$) δ 111.4 (2CH), 119.0 (2CH), 123.9 (CH), 127.3 (CH), 136.9 (C), 141.9 (CH), 146.7 (CH).

***N*-(4-Methoxyphenyl)pyrrole (1d)** [3]**.** The procedure 1 (72 h reaction time) using pyrrole (0.42 mL) and 4-iodoanisole (0.94 g) gave **1d** (eluent: heptane-CH_2Cl_2 80:20) in 46% yield as a white powder: mp 110 °C; ^{1}H NMR ($CDCl_3$) δ 3.84 (s, 3H), 6.33 (t, 2H, *J* = 2.1 Hz), 6.92-6.98 (m, 2H), 7.01 (t, 2H, *J* = 2.1 Hz), 7.30-7.34 (m, 2H); ^{13}C NMR ($CDCl_3$) δ 55.7 (CH_3), 110.0 (2CH), 114.7 (2CH), 119.8 (2CH), 122.3 (2CH), 134.6 (C), 157.7 (C).

***N*-(4-Bromophenyl)pyrrole (1e)** [4]**.** The procedure 1 (72 h reaction time) using pyrrole (0.42 mL) and 1-bromo-4-iodobenzene (1.1 g) gave **1e** (eluent: heptane-CH_2Cl_2 90:10) in 81% yield as a white powder: mp 92 °C; ^{1}H NMR ($CDCl_3$) δ 6.41 (t, 2H, *J* = 2.3 Hz), 7.09 (t, 2H, *J* = 2.3 Hz), 7.26-7.32 (m, 2H), 7.53-7.59 (m, 2H); ^{13}C NMR ($CDCl_3$) δ 111.0 (2CH), 118.7 (C), 119.2 (2CH), 121.9 (2CH), 132.6 (2CH), 139.7 (C).

***N*-(2-Thienyl)indole (2a)** [5]**.** The procedure 1 (48 h reaction time) using indole (0.70 g) and 2-iodothiophene (0.44 mL) gave **2a** (eluent: heptane-CH_2Cl_2 90:10) in 50% yield as a yellow oil: ^{1}H NMR ($CDCl_3$) δ 6.92 (dd, 1H, *J* = 3.3 and 0.6 Hz), 7.20-7.33 (m, 3H), 7.44-7.56 (m, 3H), 7.87 (d, 1H, *J* = 8.1 Hz), 7.94 (dd, 1H *J* = 7.8 and 0.9 Hz); ^{13}C NMR ($CDCl_3$) δ 104.2 (CH), 110.7 (CH), 120.3 (CH), 120.9 (CH), 121.1 (CH), 121.5 (CH), 122.9 (CH), 126.0 (CH), 129.0 (C), 129.3 (CH), 137.0 (C), 141.6 (C).

***N*-(3-Pyridyl)indole (2b)** [6]**.** The procedure 1 (48 h reaction time) using indole (0.70 g) and 3-iodopyridine (0.82 g) gave **2b** (eluent: heptane-AcOEt 80:20) in 70% yield as a yellow oil:

^{1}H NMR ($CDCl_3$) δ 6.80 (dd, 1H, J = 3.3 and 0.9 Hz), 7.24-7.35 (m, 2H), 7.36 (d, 1H, J = 3.3 Hz), 7.48 (dd, 1H, J = 8.1 and 4.8 Hz), 7.59 (d, 1H, J = 8.1 Hz), 7.75-7.79 (m, 1H), 7.85 (ddd, 1H, J = 8.1, 2.7 and 1.5 Hz), 8.65 (dd, 1H, J = 4.8 and 1.5 Hz), 8.89 (d, 1H J = 2.7 Hz); ^{13}C NMR ($CDCl_3$) δ 104.8 (CH), 109.9 (CH), 120.9 (CH), 121.4 (CH), 122.9 (CH), 124.1 (CH), 127.3 (CH), 129.4 (C), 131.4 (CH), 135.6 (C), 136.4 (C), 145.3 (CH), 147.2 (CH).

***N*-Phenylindole (2c)** [7]**.** The procedure 1 (24 h reaction time) using indole (0.70 g) and iodobenzene (0.82 g) gave **2c** (eluent: heptane-CH_2Cl_2 90:10) in 30% yield as a yellow oil: ^{1}H NMR ($CDCl_3$) δ 6.73 (dd, 1H, J = 3.3 and 0.9 Hz), 7.18-7.30 (m, 2H), 7.35-7.42 (m, 2H), 7.53-7.56 (m, 4H), 7.59-7.63 (m, 1H), 7.71-7.76 (m, 1H); ^{13}C NMR ($CDCl_3$) δ 103.7 (CH), 110.6 (CH), 120.5 (CH), 121.2 (CH), 122.5 (CH), 124.5 (2CH), 126.6 (CH), 128.1 (CH), 129.4 (C), 129.7 (2CH), 135.9 (C), 139.9 (C).

***N*-(4-Methoxyphenyl)indole (2d)** [8]**.** The procedure 1 (72 h reaction time) using indole (0.70 g) and 4-iodoanisole (0.94 g) gave **2d** (eluent: heptane-CH_2Cl_2 90:10) in 15% yield as a white powder: mp 58 °C; ^{1}H NMR ($CDCl_3$) δ 3.89 (s, 3H), 6.68 (dd, 1H, J = 3.3 and 0.9 Hz), 7.02-7.08 (m, 2H), 7.15-7.26 (m, 2H), 7.30 (d, 1H, J = 3.0 Hz), 7.40-7.46 (m, 2H), 7.46-7.51 (m, 1H), 7.69-7.73 (m, 1H); ^{13}C NMR ($CDCl_3$) δ 55.7 (CH_3), 103.0 (CH), 110.5 (CH), 114.8 (2CH), 120.2 (CH), 121.1 (CH), 122.3 (CH), 126.1 (2CH), 128.4 (CH), 129.1 (C), 133.0 (C), 136.4 (C), 158.4 (C).

***N*-(4-Bromophenyl)indole (2e)** [9]**.** The procedure 1 (56 h reaction time) using indole (0.70 g) and 1-bromo-4-iodobenzene (1.1 g) gave **2e** (eluent: heptane-CH_2Cl_2 90:10) in 60% yield as a yellow oil: ^{1}H NMR ($CDCl_3$) δ 6.74 (dd, 1H, J = 3.3 and 0.9 Hz), 7.20-7.31 (m, 2H), 7.32 (d, 1H, J = 3.3 Hz), 7.38-7.43 (m, 2H), 7.54-7.59 (m, 1H), 7.63-7.69 (m, 2H), 7.72-7.76 (m, 1H); ^{13}C NMR ($CDCl_3$) δ 104.3 (CH), 110.4 (CH), 119.8 (C), 120.7 (CH), 121.4 (CH), 122.8 (CH), 125.9 (2CH), 127.7 (CH), 129.5 (C), 132.8 (2CH), 135.8 (C), 138.9 (C). *Crystal data for **2e**.* $C_{14}H_{10}BrN$, M = 272.14, monoclinic, $P2_1/n$, a = 9.7442(7), b = 13.1654(12), c = 17.8252(17) Å, β = 93.934(4) °, V = 2281.3(3) Å^3, Z = 8, d = 1.585 g cm^{-3}, μ = 3.572 mm^{-1}. A final refinement on F^2 with 5231 unique intensities and 289 parameters converged at $\omega R(F^2)$ = 0.2092 ($R(F)$ = 0.0822) for 3568 observed reflections with $I > 2\sigma(I)$.

***N*-(4-(Trifluoromethyl)phenyl)pyrrole (1f)** [10]**.** The procedure 2 (24 h reaction time) using pyrrole (0.69 mL) and 1-iodo-4-(trifluoromethyl)benzene (1.8 mL) gave **1f** (eluent: heptane-

AcOEt 90:10) in 96% yield as a white powder: mp 112 °C; ^{1}H NMR ($CDCl_3$) δ 6.40 (t, 2H, *J* = 2.1 Hz), 7.14 (t, 2H, *J* = 2.1 Hz), 7.50 (d, 2H, *J* = 8.1 Hz), 7.69 (d, 2H, *J* = 8.4 Hz); ^{13}C NMR ($CDCl_3$) δ 111.6 (2CH), 119.2 (2CH), 120.0 (2CH), 124.2 (q, C, *J* = 270 Hz), 127.0 (q, 2CH, *J* = 3.8 Hz), 127.5 (q, C, *J* = 33 Hz), 143.3 (C).

***N*-(4-(Trifluoromethyl)phenyl)indole (2f)** [11]**.** The procedure 2 (24 h reaction time) using indole (1.2 g) and 1-iodo-4-(trifluoromethyl)benzene (1.8 mL) gave **2f** (eluent: heptane-CH_2Cl_2 70:30) in 90% yield as a white powder: mp 62 °C; ^{1}H NMR ($CDCl_3$) δ 6.76 (dd, 1H, *J* = 3.3 and 0.9 Hz), 7.21-7.33 (m, 2H), 7.38 (d, 1H, *J* = 3.3 Hz), 7.61-7.68 (m, 3H), 7.73 (dm, 1H, *J* = 7.5 Hz), 7.81 (d, 1H, *J* = 8.4 Hz); ^{13}C NMR ($CDCl_3$) δ 105.0 (CH), 110.5 (CH), 121.1 (CH), 121.6 (CH), 123.1 (CH), 124.0 (2CH), 124.1 (q, C, *J* = 271 Hz), 127.0 (q, 2CH, *J* = 3.7 Hz), 127.5 (CH), 128.3 (q, C, *J* = 32 Hz), 129.8 (C), 135.6 (C), 142.9 (C).

***N*-(5-iodo-2-thienyl)pyrrole (3a).** The procedure 3 using *N*-(2-thienyl)pyrrole (**1a**, 0.15 g) gave **3a** (eluent: heptane-AcOEt 80:20) in 97% yield as a beige powder: mp < 50 °C; IR (ATR): 678, 722, 747, 769, 784, 810, 881, 937, 995, 1018, 1047, 1066, 1109, 1213, 1228, 1252, 1267, 1283, 1310, 1480, 1511, 1558, 1723, 2964 cm^{-1}; ^{1}H NMR ($CDCl_3$) δ 6.37 (t, 2H, *J* = 2.1 Hz), 6.62 (d, 1H, *J* = 3.9 Hz), 6.97 (t, 2H, *J* = 2.1 Hz), 7.14 (d, 1H, *J* = 3.9 Hz); ^{13}C NMR ($CDCl_3$) δ 66.2 (C), 110.9 (2CH), 117.0 (CH), 121.0 (2CH), 135.9 (CH), 148.5 (C); MS (ESI): calcd for C_8H_6INS $[M]^+$ 275, found 275.

***N*-(5-Iodo-2-thienyl)indole (4a).** The procedure 3 using *N*-(2-thienyl)indole (**2a**, 0.20 g) gave **4a** (eluent: heptane-AcOEt 80:20) in 91% yield as a yellow oil; IR (ATR): 717, 739, 761, 793, 891, 944, 1014, 1131, 1196, 1213, 1226, 1285, 1306, 1331, 1457, 1475, 1516, 1550, 3051 cm^{-1}; ^{1}H NMR ($CDCl_3$) δ 6.79 (dd, 1H, *J* = 3.3 and 0.9 Hz), 6.83 (d, 1H, *J* = 3.9 Hz), 7.30-7.44 (m, 4H), 7.70 (dm, 1H, *J* = 8.4 Hz), 7.80 (dm, 1H, *J* = 8.0 Hz); ^{13}C NMR ($CDCl_3$) δ 68.9 (C), 104.8 (CH), 110.6 (CH), 121.2 (CH), 121.2 (CH), 121.9 (CH), 123.2 (CH), 128.8 (CH), 129.1 (C), 135.8 (CH), 136.8 (C), 146.2 (C); MS (ESI): calcd for $C_{12}H_8INS$ $[M]^+$ 325, found 325.

***N*-(2-Iodo-3-pyridyl)pyrrole (3b).** The procedure 4 using *N*-(3-pyridyl)pyrrole (**1b**, 72 mg) gave **3b** (eluent: heptane-AcOEt 50:50) in 59% yield as a beige powder: mp 104-106 °C; IR (ATR): 705, 733, 815, 923, 1010, 1047, 1068, 1083, 1109, 1209, 1237, 1332, 1383, 1397, 1461, 1491, 1556, 3125 cm^{-1}; ^{1}H NMR ($CDCl_3$) δ 6.34 (t, 2H, *J* = 2.2 Hz), 6.80 (t, 2H, *J* = 2.2 Hz), 7.33 (dd, 1H, *J* = 7.8 and 4.5 Hz), 7.50 (dd, 1H, *J* = 7.8 and 1.8 Hz), 8.37 (dd, 1H, *J* =

4.5 and 1.8 Hz); ^{13}C NMR ($CDCl_3$) δ 110.1 (2CH), 119.9 (C), 122.1 (2CH), 123.1 (CH), 134.7 (CH), 141.8 (C), 149.5 (CH); MS (ESI): calcd for $C_9H_7IN_2$ $[M]^+$ 270, found 270.

***N*-(2-Iodo-3-pyridyl)indole (4b).** The procedure 4 using *N*-(3-pyridyl)indole (**2b**, 0.19 g) gave **4b** (eluent: heptane-AcOEt 80:20) in 71% yield as a yellow oil; IR (ATR): 678, 709, 726, 744, 768, 811, 884, 1017, 1046, 1140, 1187, 1213, 1227, 1250, 1267, 1288, 1308, 1332, 1399, 1438, 1460, 1510, 1566, 2965, 3050 cm^{-1}; ^1H NMR ($CDCl_3$) δ 6.75 (dd, 1H, J = 3.3 and 0.9 Hz), 7.01-7.24 (m, 4H), 7.42 (dd, 1H, J = 7.8 and 4.5 Hz), 7.63 (dd, 1H, J = 7.8 and 1.8 Hz), 7.70-7.74 (m, 1H), 8.49 (dd, 1H, J = 4.5 and 1.8 Hz); ^{13}C NMR ($CDCl_3$) δ 104.3 (CH), 110.4 (CH), 120.8 (CH), 121.3 (CH), 121.9 (C), 122.8 (CH), 123.4 (CH), 128.4 (CH), 128.7 (C), 136.4 (CH), 136.8 (C), 140.2 (C), 150.2 (CH); MS (ESI): calcd for $C_{13}H_9IN_2$ $[M]^+$ 320, found 320.

2-Iodo-*N*-(3-iodo-4-methoxyphenyl)indole (4d'). The procedure 4 using *N*-(4-methoxyphenyl)-1*H*-indole (**2d**, 0.11 g) gave **4d'** (eluent: heptane-AcOEt 80:20) in 15% yield as a beige powder: mp 166 °C; IR (ATR): 681, 731, 745, 782, 817, 906, 1018, 1046, 1147, 1212, 1248, 1265, 1286, 1309, 1440, 1464, 1490, 1564, 1595, 2838, 2939, 3061 cm^{-1}; ^1H NMR ($CDCl_3$) δ 3.98 (s, 3H), 6.93 (d, 1H, J = 0.6 Hz), 6.95 (d, 1H, J = 8.7 Hz), 7.08-7.13 (m, 3H), 7.32 (dd, 1H, J = 8.7 and 2.4 Hz), 7.56-7.59 (m, 1H), 7.78 (d, 1H, J = 2.7 Hz); ^{13}C NMR ($CDCl_3$) δ 56.8 (CH_3), 84.5 (C), 85.6 (C), 110.6 (CH), 110.8 (CH), 113.7 (CH), 119.5 (CH), 120.6 (CH), 122.6 (CH), 129.7 (C), 130.2 (CH), 132.7 (C), 139.6 (C), 139.8 (CH), 158.4 (C); MS (ESI): calcd for $C_{15}H_{11}I_2NO$ $[M]^+$ 475, found 475.

***N*-(4-Bromo-3-iodophenyl)pyrrole (3e).** The procedure 4 using *N*-(4-bromophenyl)pyrrole (**1e**, 0.11 g) gave **3e** (eluent: heptane-AcOEt 80:20) in 58% yield as a yellow powder: mp 64-66 °C; IR (ATR): 656, 723, 812, 867, 928, 1007, 1021, 1067, 1123, 1264, 1328, 1397, 1456, 1488, 1525, 1560, 1580, 2927, 3103 cm^{-1}; ^1H NMR ($CDCl_3$) δ 6.38 (t, 2H, J = 2.2 Hz), 7.03 (t, 2H, J = 2.1 Hz), 7.23 (dd, 1H, J = 8.7 and 2.7 Hz), 7.62 (d, 1H, J = 8.7 Hz), 7.89 (d, 1H, J = 2.7 Hz); ^{13}C NMR ($CDCl_3$) δ 101.9 (C), 111.4 (2CH), 119.2 (2CH), 121.3 (CH), 126.1 (C), 131.7 (CH), 133.1 (CH), 140.2 (C). *Crystal data for **3e**.* $C_{10}H_7BrIN$, M = 347.98, monoclinic, $P2_1/n$, a = 10.0665(5), b = 5.9895(4), c = 17.5155(10) Å, β = 103.942(3) °, V = 1024.96(10) Å^3, Z = 4, d = 2.255 g cm^{-3}, μ = 6.974 mm^{-1}. A final refinement on F^2 with 2335 unique intensities and 118 parameters converged at $\omega R(F^2)$ = 0.0789 ($R(F)$ = 0.032) for 1971 observed reflections with $I > 2\sigma(I)$.

***N*-(4-Bromo-3-iodophenyl)indole (4e).** The procedure 4 using *N*-(4-bromophenyl)indole (**2e**, 0.14 g) gave **4e** (eluent: heptane-AcOEt 80:20) in 57% yield as a yellow oil: IR (ATR): 694, 738, 761, 820, 883, 907, 963, 1009, 1072, 1100, 1133, 1211, 1232, 1298, 1333, 1465, 1490, 1516, 1579, 1709, 1895, 2929, 3051 cm^{-1}; ^1H NMR ($CDCl_3$) δ 6.70 (dd, 1H, J = 3.3 and 0.6 Hz), 7.16-7.29 (m, 2H), 7.27 (d, 1H, J = 3.3 Hz), 7.38 (dd, 1H, J = 8.4 and 2.4 Hz), 7.52 (dd, 1H, J = 8.4 and 0.9 Hz), 7.68 (dd, 1H, J = 7.2 and 1.8 Hz), 7.74 (d, 1H, J = 8.7 Hz), 8.02 (d, 1H, J = 2.4 Hz); ^{13}C NMR ($CDCl_3$) δ 101.9 (C), 104.7 (CH), 110.2 (CH), 120.9 (CH), 121.4 (CH), 122.9 (CH), 125.0 (CH), 127.0 (C), 127.3 (CH), 129.5 (C), 133.2 (CH), 135.3 (CH), 135.4 (C), 139.3 (C); MS (ESI): calcd for $C_{14}H_9BrIN$ $[M]^+$ 397, found 397.

2-Iodo-*N*-phenylpyrrole (3c). The procedure 5 using commercial *N*-phenylpyrrole (**1c**, 95 mg) gave **3c** (eluent: heptane-AcOEt 80:20) in 86% yield as a yellow oil; IR (ATR): 692, 710, 761, 782, 875, 940, 1033, 1072, 1116, 1299, 1320, 1388, 1431, 1457, 1496, 1513, 1597, 2925, 3047, 3103 cm^{-1}; ^1H NMR ($CDCl_3$) δ 6.19 (dd, 1H, J = 3.6 and 3.0 Hz), 6.41 (dd, 1H, J = 3.6 and 1.8 Hz), 6.87 (dd, 1H, J = 3.0 and 1.8 Hz), 7.19-7.32 (m, 5H); ^{13}C NMR ($CDCl_3$) δ 69.1 (C), 111.8 (CH), 120.4 (CH), 125.7 (CH), 126.8 (2CH), 127.8 (CH), 128.8 (2CH), 140.8 (C). The analyses are similar to those previously reported [12].

2-Iodo-*N*-phenylindole (4c). The procedure 5 using *N*-phenylindole (**2c**, 0.13 g) gave **4c** (eluent: heptane-AcOEt 80:20), after recrystallization from pentane, in 92% yield as a yellow powder: mp 88 °C; IR (ATR): 678, 691, 746, 763, 780, 809, 962, 1016, 1046, 1070, 1139, 1212, 1227, 1251, 1267, 1289, 1307, 1436, 1457, 1494, 1594, 2924, 3043 cm^{-1}; ^1H NMR ($CDCl_3$) δ 6.99 (s, 1H), 7.11-7.18 (m, 3H), 7.37-7.42 (m, 2H), 7.53-7.64 (m, 4H); ^{13}C NMR ($CDCl_3$) δ 83.9 (C), 110.9 (CH), 113.8 (CH), 119.5 (CH), 120.5 (CH), 122.4 (CH), 128.7 (CH), 129.1 (2CH), 129.4 (2CH), 129.7 (C), 138.8 (C), 139.5 (C). *Crystal data for **4c**.* $C_{14}H_{10}IN$, M = 319.13, monoclinic, $C\,c$, a = 7.0800(3), b = 20.6660(10), c = 8.2442(5) Å, β = 100.632(2) °, V = 1185.54(10) $Å^3$, Z = 4, d = 1.788 g cm^{-3}, μ = 2.671 mm^{-1}. A final refinement on F^2 with 2183 unique intensities and 145 parameters converged at $\omega R(F^2)$ = 0.0563 ($R(F)$ = 0.0233) for 2139 observed reflections with $I > 2\sigma(I)$.

***N*-(3-Iodo-4-methoxyphenyl)pyrrole (3d).** The procedure 6 using *N*-(4-methoxyphenyl)pyrrole (**1d**, 0.12 g) gave **3d** (eluent: heptane-AcOEt 80:20) in 97% yield as a white powder: mp 126-128 °C; IR (ATR): 670, 739, 808, 885, 932, 1011, 1024, 1047, 1070, 1125, 1181, 1242, 1259, 1294, 1332, 1413, 1440, 1456, 1499, 1575, 1600, 2835, 2958, 3128

cm^{-1}; ^{1}H NMR ($CDCl_3$) δ 3.90 (s, 3H), 6.35 (t, 2H, J = 2.3 Hz), 6.85 (d, 1H, J = 8.7 Hz), 6.99 (t, 2H, J = 2.3 Hz), 7.34 (dd, 1H, J = 8.7 and 2.4 Hz), 7.83 (d, 1H, J = 2.7 Hz); ^{13}C NMR ($CDCl_3$) δ 56.7 (CH_3), 86.2 (C), 110.4 (2CH), 111.1 (CH), 119.6 (2CH), 121.9 (CH), 132.0 (CH), 135.4 (C), 156.3 (C). *Crystal data for **3d**.* $C_{11}H_{10}INO$, M = 299.10, monoclinic, $P\ 2_1/n$, a = 9.9461(4), b = 6.9825(3), c = 15.2049(8) Å, β = 92.314(2) °, V = 1055.10(8) Å^3, Z = 4, d = 1.883 g cm^{-3}, μ = 3.001 mm^{-1}. A final refinement on F^2 with 2409 unique intensities and 128 parameters converged at $\omega R(F^2)$ = 0.0548 ($R(F)$ = 0.0253) for 2149 observed reflections with $I > 2\sigma(I)$.

***N*-(3-Iodo-4-methoxyphenyl)indole (4d).** The procedure 6 using *N*-(4-methoxyphenyl)indole (**2d**, 0.15 g) gave **4d** (eluent: heptane-AcOEt 80:20), after recrystallization from pentane, in 71% yield as a beige powder: mp 98-100 °C; IR (ATR): 678, 726, 747, 768, 810, 885, 968, 1017, 1046, 1112, 1139, 1186, 1215, 1227, 1250, 1267, 1287, 1334, 1459, 1492, 1510, 1597, 2965 cm^{-1}; ^{1}H NMR ($CDCl_3$) δ 3.96 (s, 3H), 6.68 (dd, 1H, J = 3.3 and 0.9 Hz), 6.94 (d, 1H, J = 8.7 Hz), 7.16-7.27 (m, 2H), 7.26 (d, 1H, J = 2.7 Hz), 7.44-7.48 (m, 2H), 7.68-7.72 (m, 1H), 7.93 (d, 1H, J = 2.7 Hz); ^{13}C NMR ($CDCl_3$) δ 56.8 (CH_3), 86.2 (C), 103.5 (CH), 110.3 (CH), 111.1 (CH), 120.5 (CH), 121.2 (CH), 122.5 (CH), 125.8 (CH), 128.1 (CH), 129.1 (C), 134.0 (C), 135.8 (CH), 136.3 (C), 157.0 (C); MS (ESI): calcd for $C_{15}H_{12}INO$ $[M]^+$ 349, found 349.

***N*-(3-(1-Pyrrolyl)-2-pyridyl)imidazole (5b).** The procedure 7 using *N*-(2-iodo-3-pyridyl)pyrrole (**3b**, 0.27 g) gave **5b** (eluent: AcOEt-heptane 80:20) in 85% yield as a yellow oil: IR (ATR): 655, 728, 765, 810, 923, 972, 1013, 1048, 1072, 1093, 1213, 1239, 1260, 1302, 1335, 1446, 1493, 1582, 3119 cm^{-1}; ^{1}H NMR ($CDCl_3$) δ 6.31 (t, 2H, J = 2.1 Hz), 6.61 (t, 2H, J = 2.1 Hz), 6.82 (s, 1H), 6.97 (s, 1H), 7.34 (dd, 1H, J = 7.8 and 4.8 Hz), 7.41 (s, 1H), 7.77 (dd, 1H, J = 8.1 and 1.7 Hz), 8.47 (dd, 1H, J = 4.8 and 1.7 Hz); ^{13}C NMR ($CDCl_3$) δ 111.4 (2CH), 117.5 (CH), 121.1 (2CH), 122.7 (CH), 128.3 (C), 129.6 (CH), 136.1 (CH), 137.3 (CH), 144.8 (C), 147.9 (CH); MS (ESI): calcd for $C_{12}H_{10}N_4$ $[M]^+$ 210, found 210.

***N*-(2-Methoxy-5-(1-pyrrolyl)phenyl)imidazole (5d).** The procedure 7 using *N*-(3-iodo-4-methoxyphenyl)pyrrole (**3d**, 0.30 g) gave **5d** (eluent: AcOEt-heptane 80:20) in 67% yield as a beige powder: mp 130 °C; IR (ATR): 659, 700, 723, 813, 985, 1019, 1032, 1055, 1070, 1247, 1263, 1281, 1464, 1496, 1520, 2841, 2937, 3109 cm^{-1}; ^{1}H NMR ($CDCl_3$) δ 3.87 (s, 3H), 6.34 (t, 2H, J = 2.1 Hz), 7.01 (t, 2H, J = 2.1 Hz), 7.10 (d, 1H, J = 8.7 Hz), 7.19 (s, 1H), 7.23 (s, 1H), 7.32 (d, 1H, J = 2.7 Hz), 7.37 (dd, 1H, J = 8.7 and 2.7 Hz), 7.84 (s, 1H); ^{13}C NMR

($CDCl_3$) δ 56.3 (CH_3), 110.7 (2CH), 113.3 (CH), 118.3 (CH), 119.6 (2CH), 120.2 (CH), 120.9 (CH), 127.0 (C), 129.0 (CH), 134.7 (C), 137.8 (CH), 150.4 (C). *Crystal data for* ***5d***. $C_{14}H_{13}N_3O$, M = 239.27, monoclinic, $P\ 2_1/n$, a = 11.4222(6), b = 5.4888(3), c = 19.7338(12) Å, β = 100.562(2) °, V = 1216.23(12) Å^3, Z = 4, d = 1.307 g cm^{-3}, μ = 0.086 mm^{-1}. A final refinement on F^2 with 2777 unique intensities and 164 parameters converged at $\omega R(F^2)$ = 0.101 ($R(F)$ = 0.0404) for 2281 observed reflections with $I > 2\sigma(I)$.

***N*-(2-Methoxy-5-(1-indolyl)phenyl)imidazole (6d).** The procedure 7 using *N*-(3-iodo-4-methoxyphenyl)indole (**4d**, 0.35 g) gave **6d** (eluent: AcOEt-heptane 80:20) in 40% yield as a yellow oil: IR (ATR): 659, 704, 737, 762, 817, 884, 1022, 1055, 1093, 1106, 1134, 1212, 1226, 1251, 1286, 1457, 1491, 1519, 1591, 1609, 2841, 2932, 3051 cm^{-1}; ^{1}H NMR ($CDCl_3$) δ 3.93 (s, 3H), 6.69 (d, 1H, J = 2.7 Hz), 7.17-7.30 (m, 6H), 7.44-7.51 (m, 3H), 7.69 (d, 1H, J = 7.2 Hz), 7.84 (br s, 1H); ^{13}C NMR ($CDCl_3$) δ 56.4 (CH_3), 103.9 (CH), 110.1 (CH), 113.3 (CH), 120.2 (CH), 120.6 (CH), 121.4 (CH), 121.8 (CH), 122.7 (CH), 124.9 (CH), 128.0 (CH), 128.0 (C), 129.0 (CH), 129.2 (C), 133.3 (C), 136.1 (C), 137.8 (CH), 151.1 (C); MS (ESI): calcd for $C_{18}H_{15}N_3O$ $[M]^+$ 289, found 289.

1. Reddy, V. P.; Kumar, A. V.; Rao, K. R. *Tetrahedron Lett.* **2011**, *52*, 777-780. doi:10.1016/j.tetlet.2010.12.016
2 Teo, Y.-C.; Yong, F.-F.; Sim, S. *Tetrahedron* **2013**, *69*, 7279-7284. doi:10.1016/j.tet.2013.06.095
3 Panda, N.; Jena, A. K.; Mohapatra, S.; Rout, S. R. *Tetrahedron Lett.* **2011**, *52*, 1924-1927. doi:10.1016/j.tetlet.2011.02.050
4 Jiang, D.; Fu, H.; Jiang, Y.; Zhao, Y. *J. Org. Chem.* **2007**, *72*, 672-674. doi:10.1021/jo062060e
5 Tang, B.-X.; Guo, S.-M.; Zhang, M.-B.; Li, J.-H. *Synthesis* **2008**, 1707-1716. doi:10.1055/s-2008-1067014
6 Romero, M.; Harrak, Y.; Basset, J.; Ginet, L.; Constans, P.; Pujol, M. D. *Tetrahedron* **2006**, *62*, 9010-9016. doi:10.1016/j.tet.2006.07.011
7 Kidwai, M.; Mishra, N. K.; Bhardwaj, S.; Jahan, A.; Kumar, A.; Mozumdar, S. *ChemCatChem* **2010**, *2*, 1312-1317. doi:10.1002/cctc.201000062
8 Rao, R. K.; Naidu, A. B.; Jaseer, E. A.; Sekar, G. *Tetrahedron* **2009**, *65*, 4619-4624. doi:10.1016/j.tet.2009.03.061
9 Rao, H.; Jin, Y.; Fu, H.; Jiang, Y.; Zhao, Y. *Chem. Eur. J.* **2006**, *12*, 3636-3646. doi:10.1002/chem.200501473
10 Ma, H.-C.; Jiang, X.-Z. *J. Org. Chem.* **2007**, *72*, 8943-8946. doi:10.1021/jo7015983
11 Bellina, F.; Calandri, C.; Cauteruccio, S.; Rossi, R. *Eur. J. Org. Chem.* **2007**, 2147-2151. doi:10.1002/ejoc.200601084
12 Seggio, A.; Lannou, M. I.; Chevallier, F.; Nobuto, D.; Uchiyama, M.; Golhen, S.; Roisnel, T.; Mongin, F. *Chem. Eur. J.* **2007**, *13*, 9982-9989. doi:10.1002/chem.200700608

Compound 3a

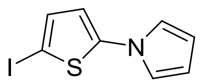

^{1}H NMR (300 MHz, $CDCl_3$)

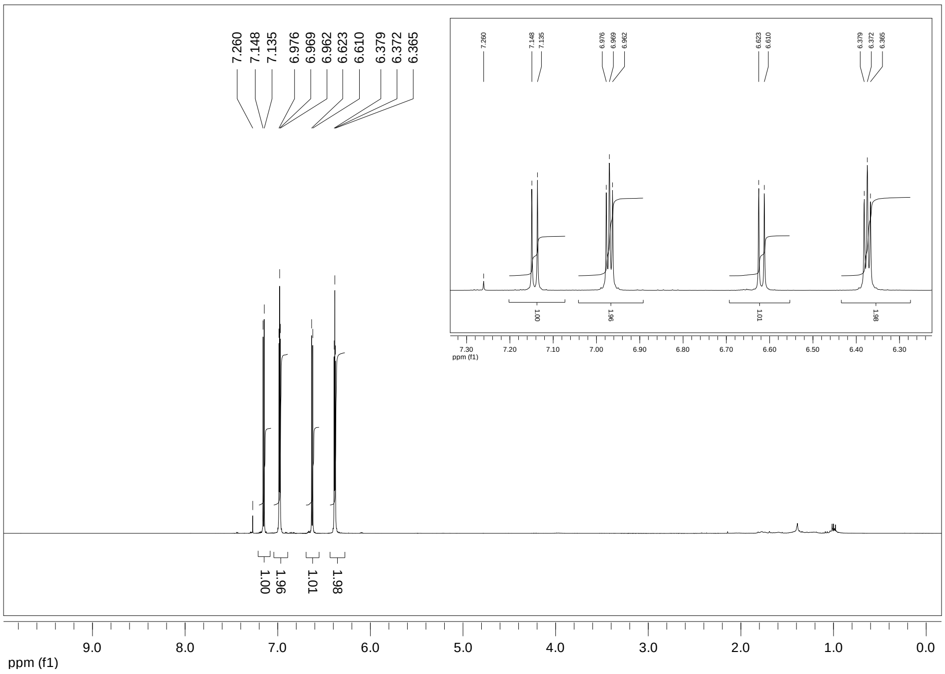

^{13}C NMR (75 MHz, $CDCl_3$)

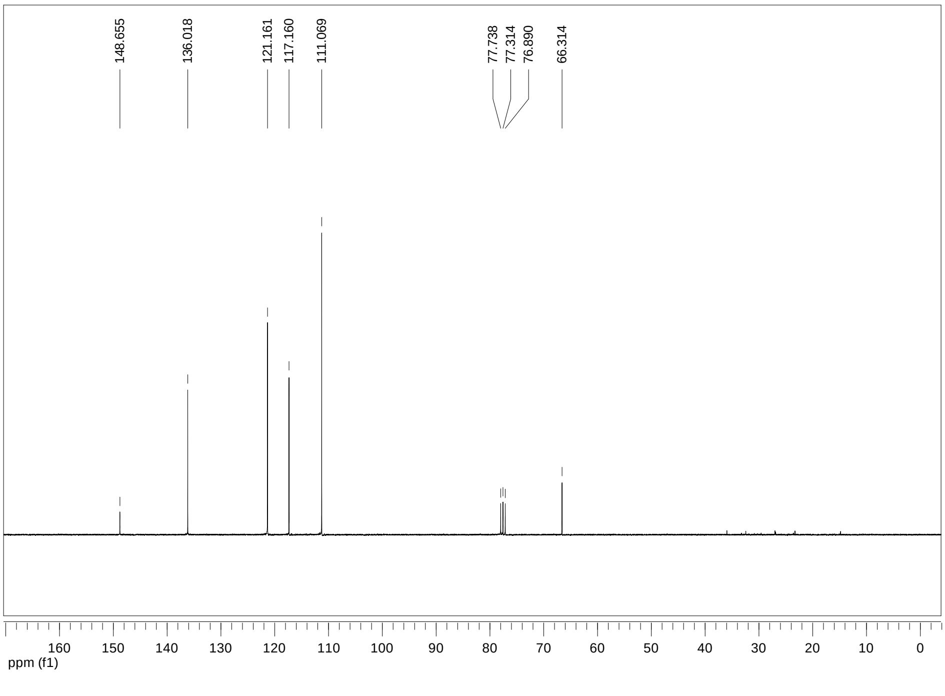

Compound 4a

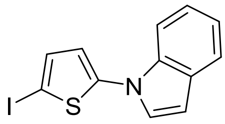

^{1}H NMR (300 MHz, $CDCl_3$)

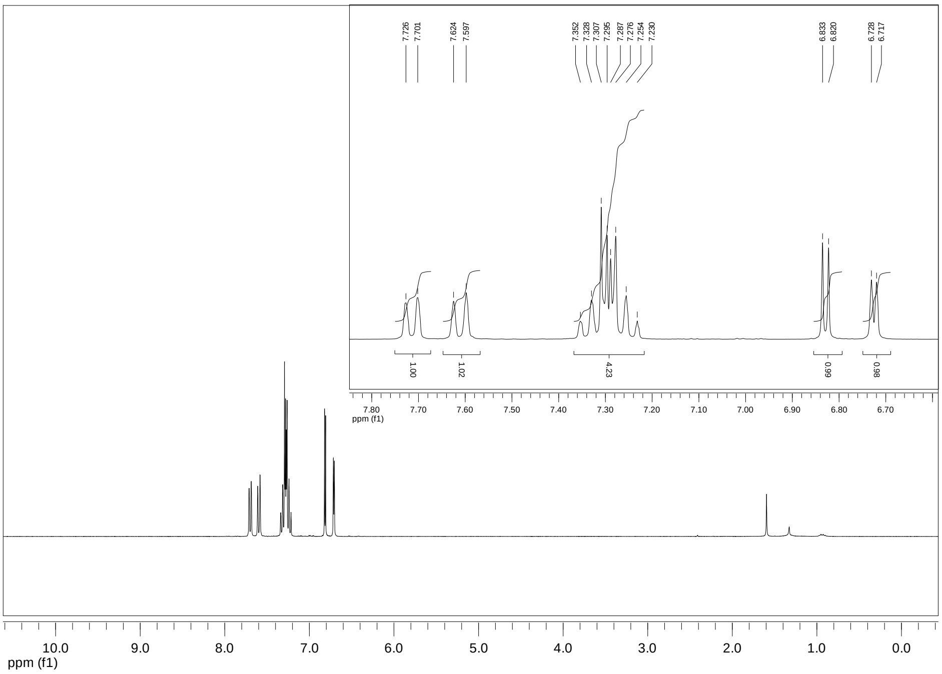

^{13}C NMR (75 MHz, $CDCl_3$)

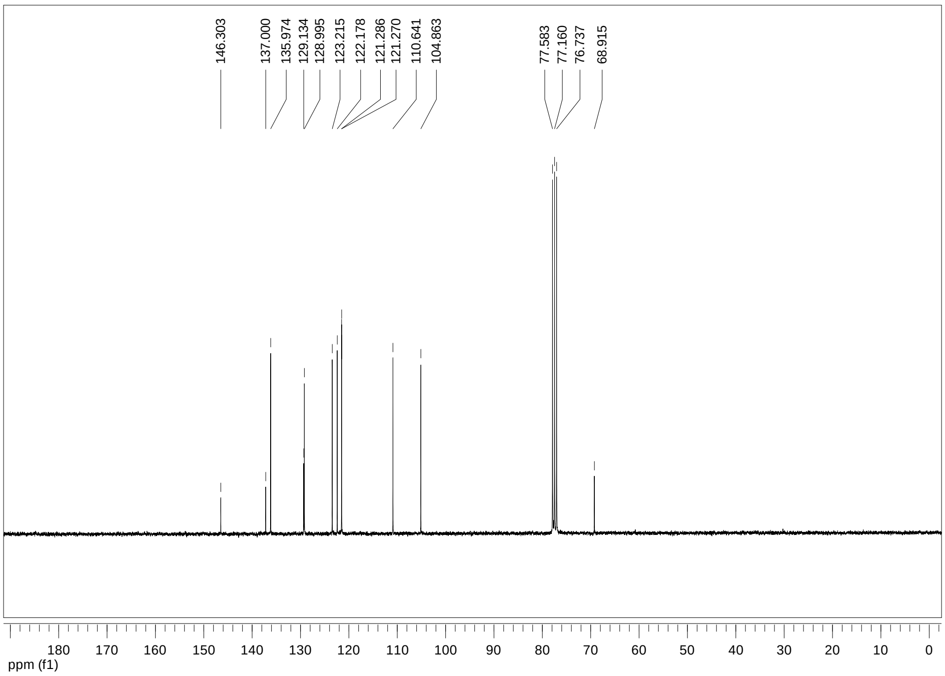

Compound 3b

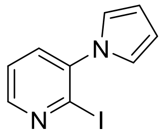

^{1}H NMR (300 MHz, $CDCl_3$)

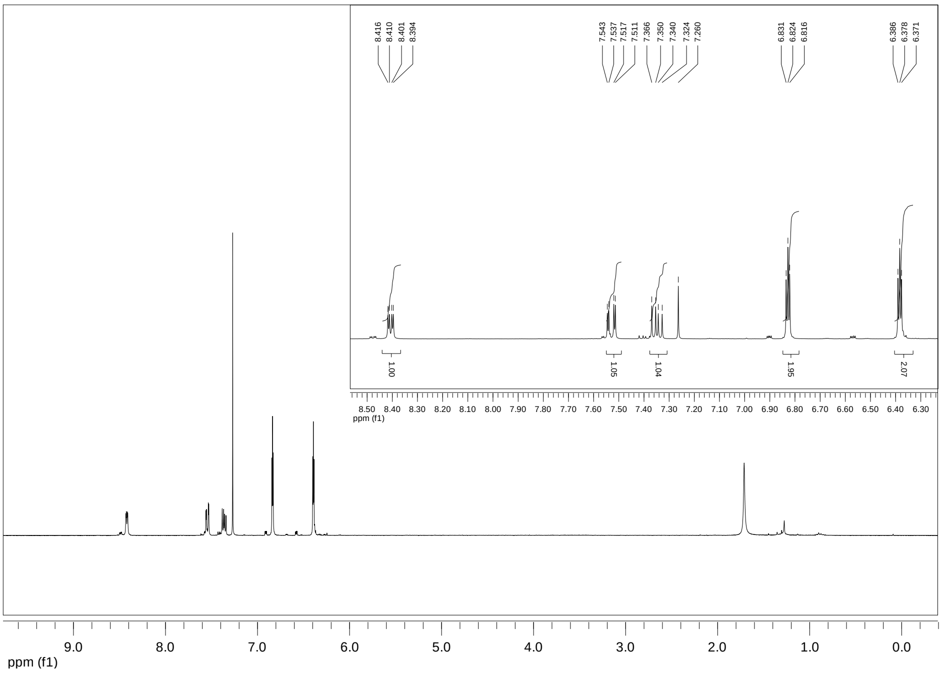

^{13}C NMR (75 MHz, $CDCl_3$)

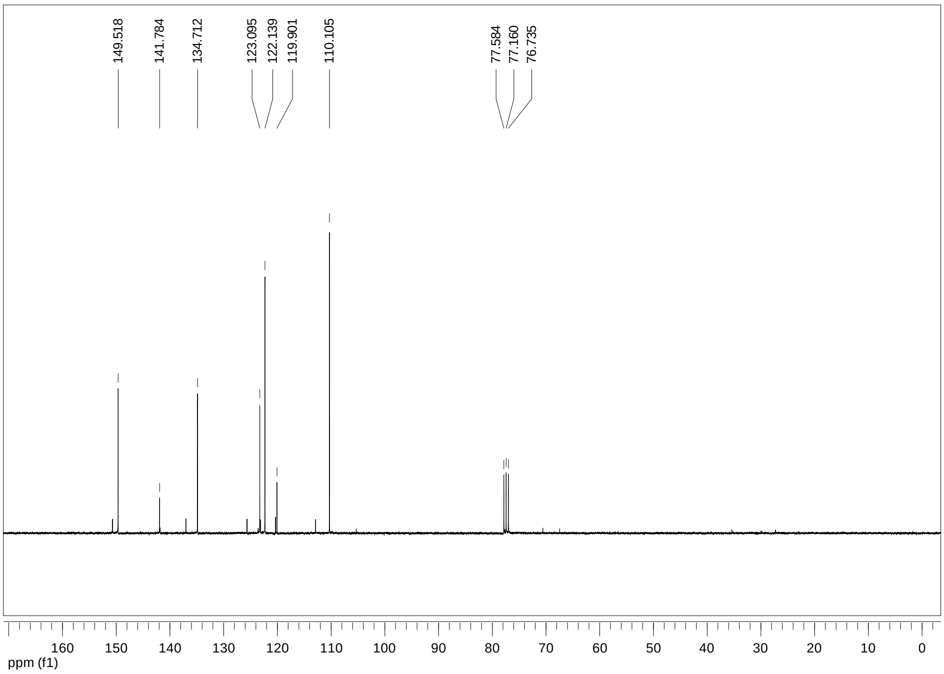

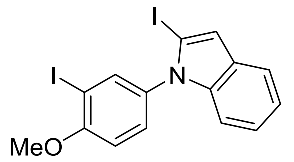

Compound 4d’

^{1}H NMR (300 MHz, $CDCl_3$)

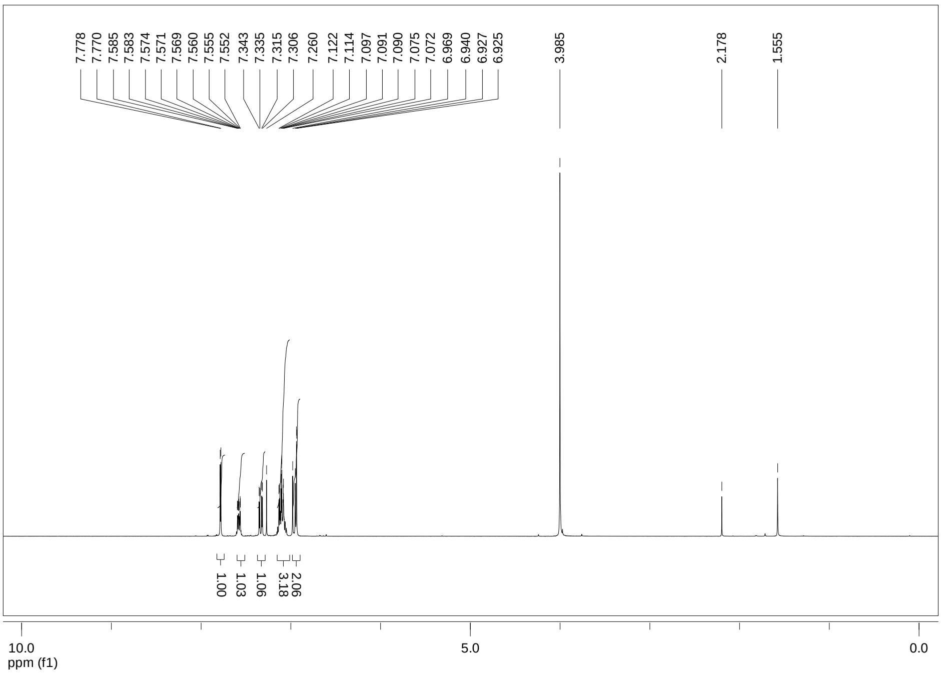

^{13}C NMR (75 MHz, $CDCl_3$)

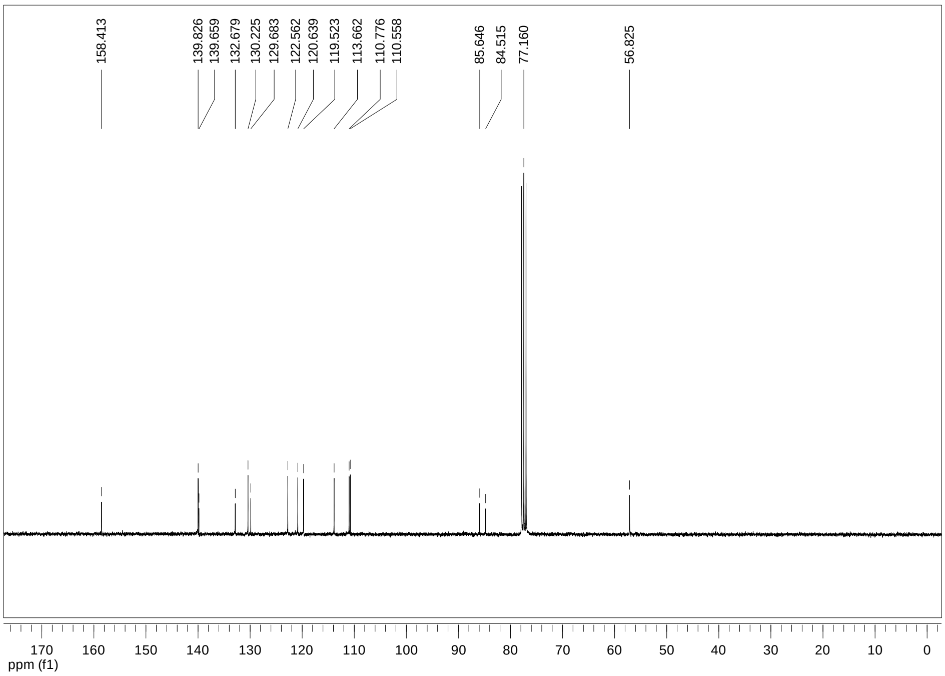

Compound 3e

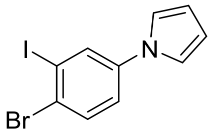

^{1}H NMR (300 MHz, $CDCl_3$)

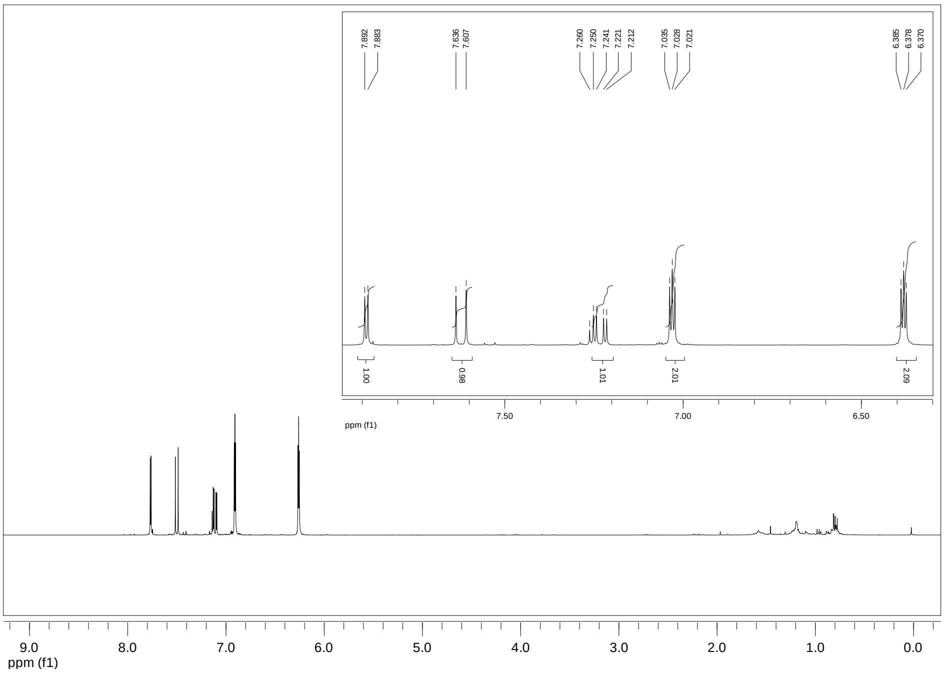

^{13}C NMR (75 MHz, $CDCl_3$)

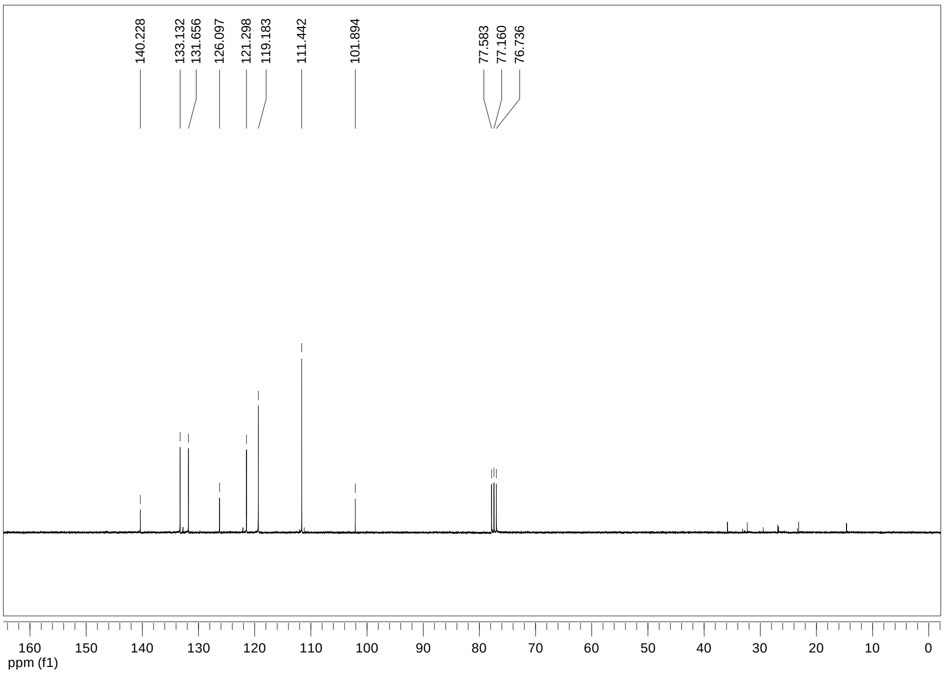

Compound 4e

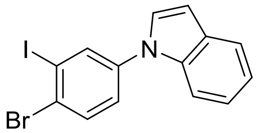

^{1}H NMR (300 MHz, $CDCl_3$)

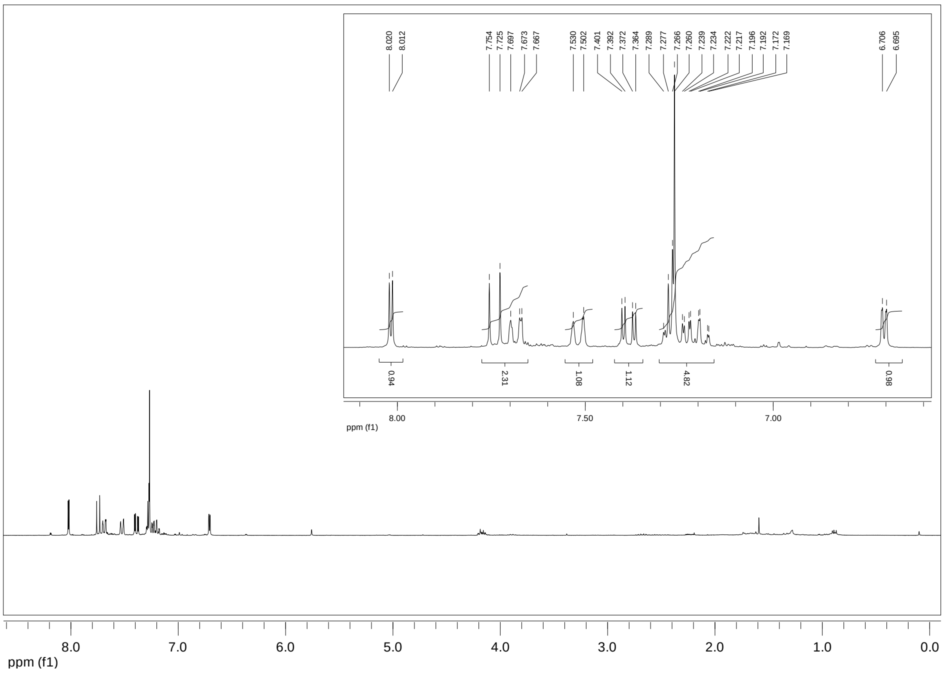

^{13}C NMR (75 MHz, $CDCl_3$)

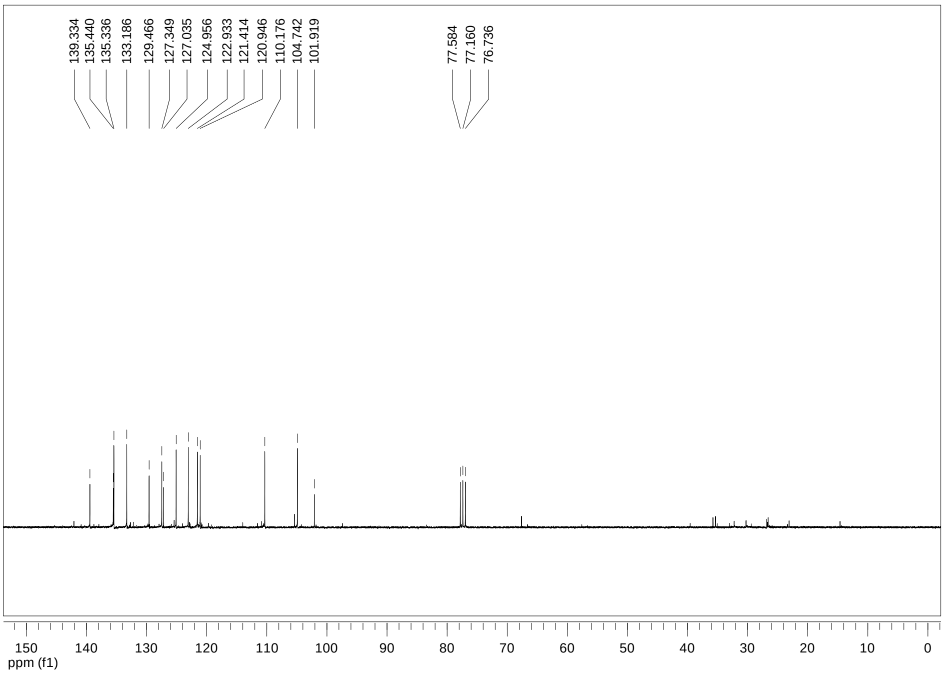

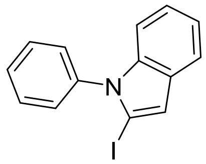

Compound 4c

^{1}H NMR (300 MHz, $CDCl_3$)

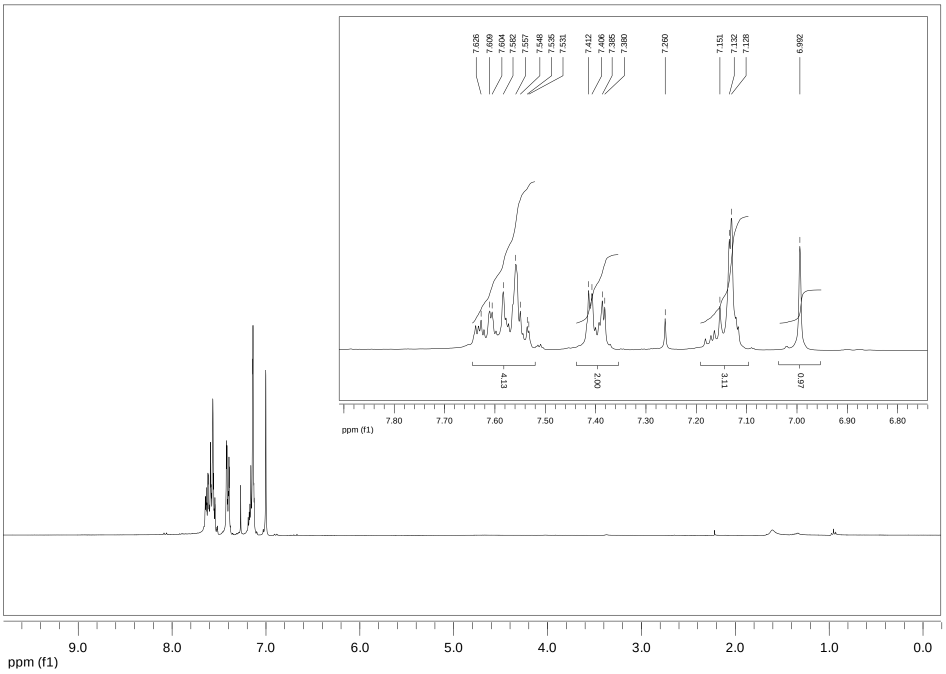

^{13}C NMR (75 MHz, $CDCl_3$)

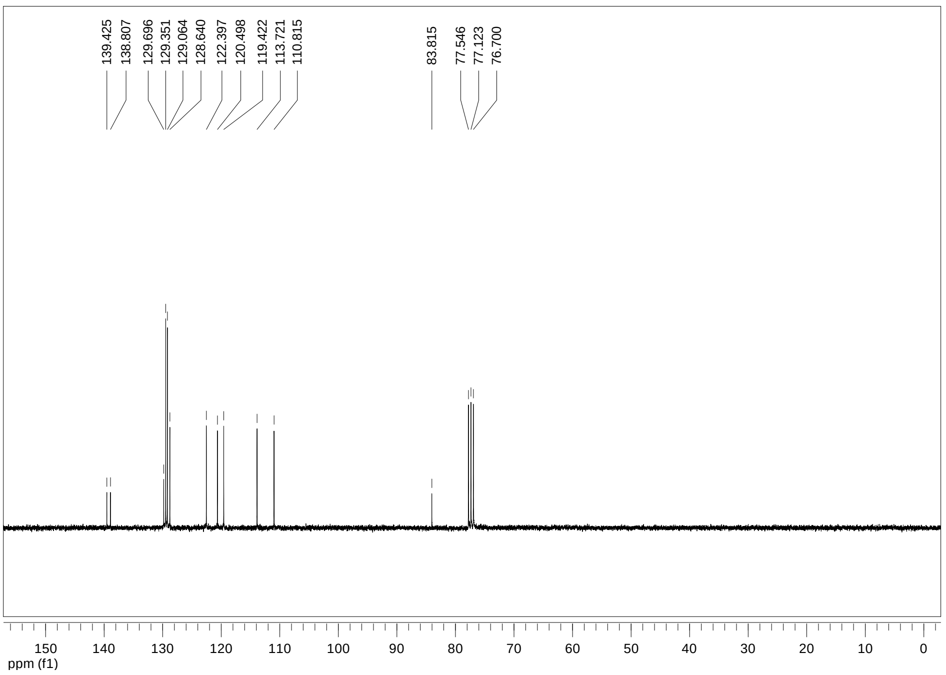

Compound 3d

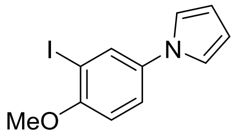

^{1}H NMR (300 MHz, $CDCl_3$)

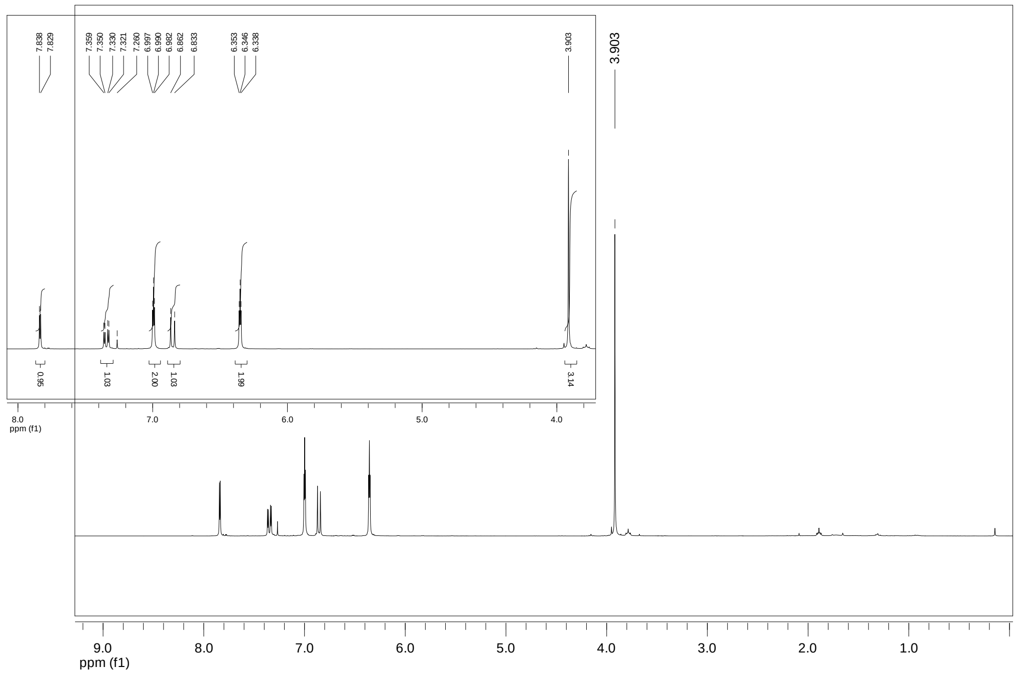

^{13}C NMR (75 MHz, $CDCl_3$)

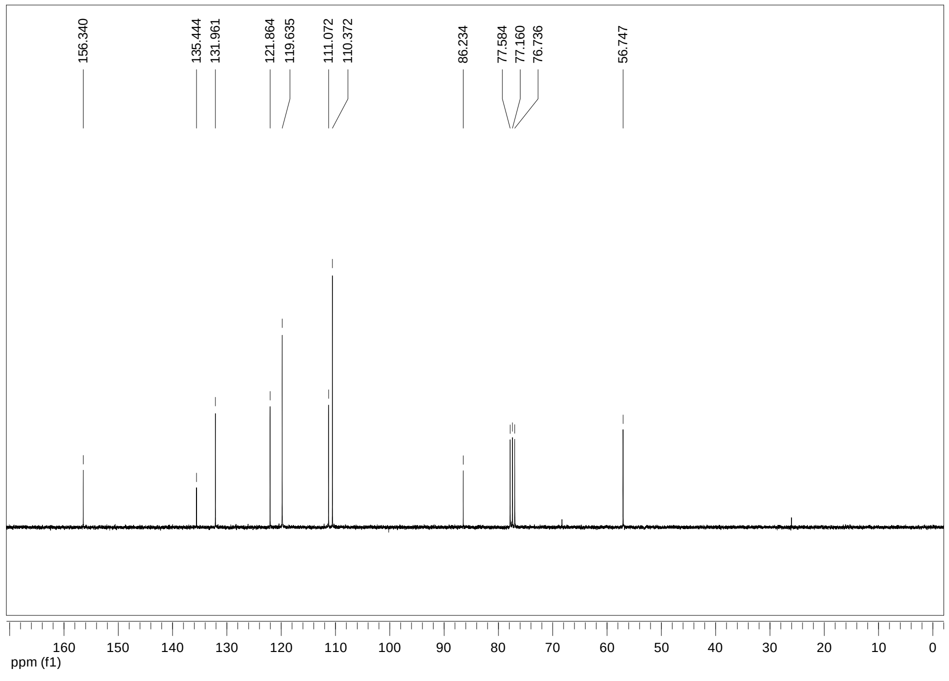

Compound 4d

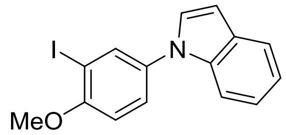

^{1}H NMR (300 MHz, $CDCl_3$)

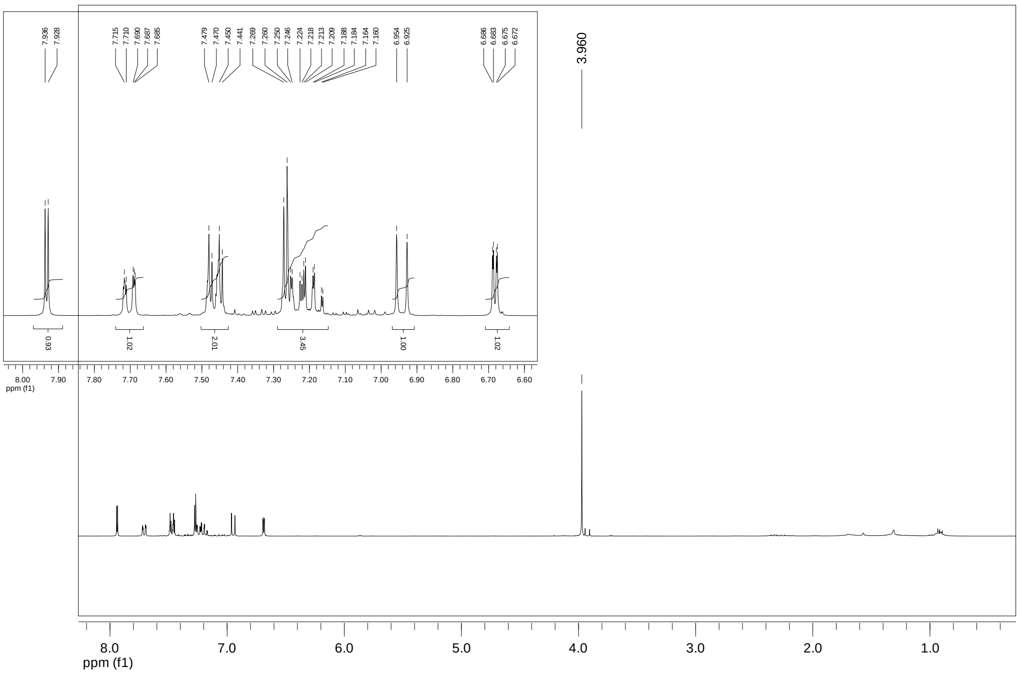

^{13}C NMR (75 MHz, $CDCl_3$)

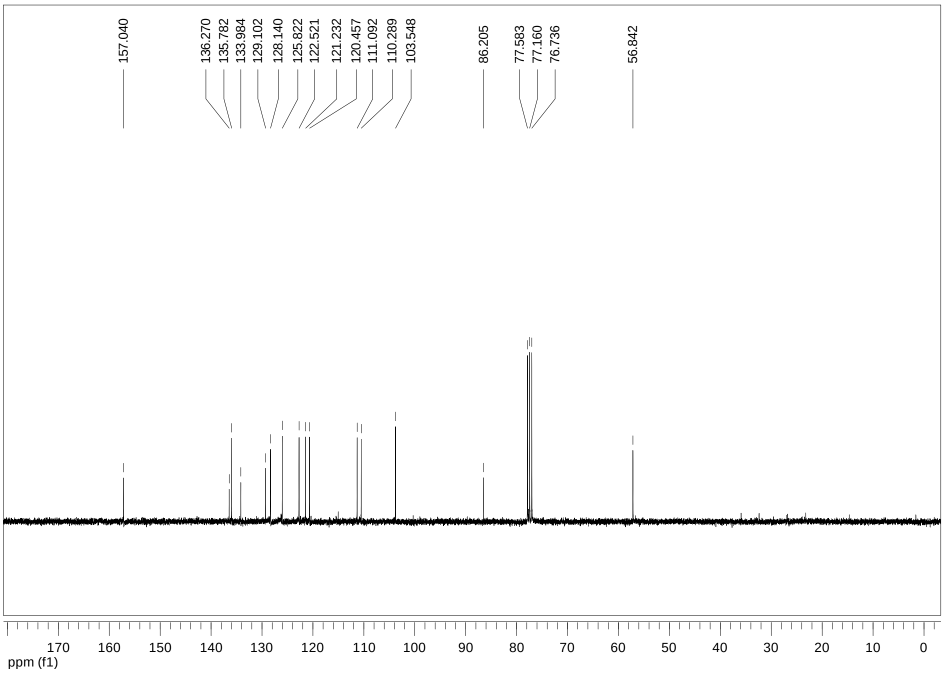

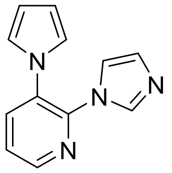

Compound 5b

^{1}H NMR (300 MHz, $CDCl_3$)

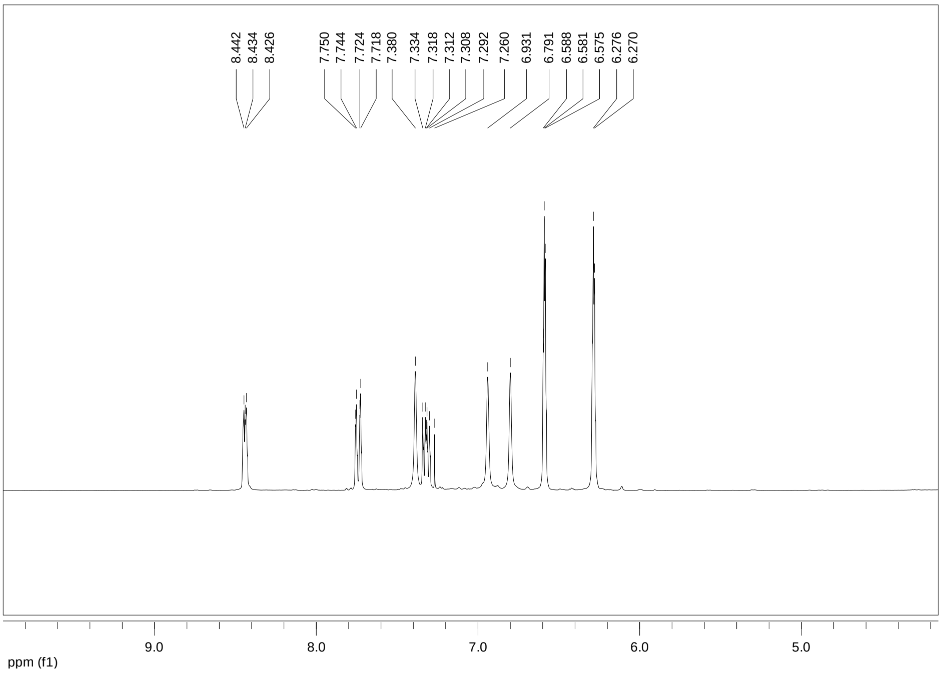

^{13}C NMR (75 MHz, $CDCl_3$)

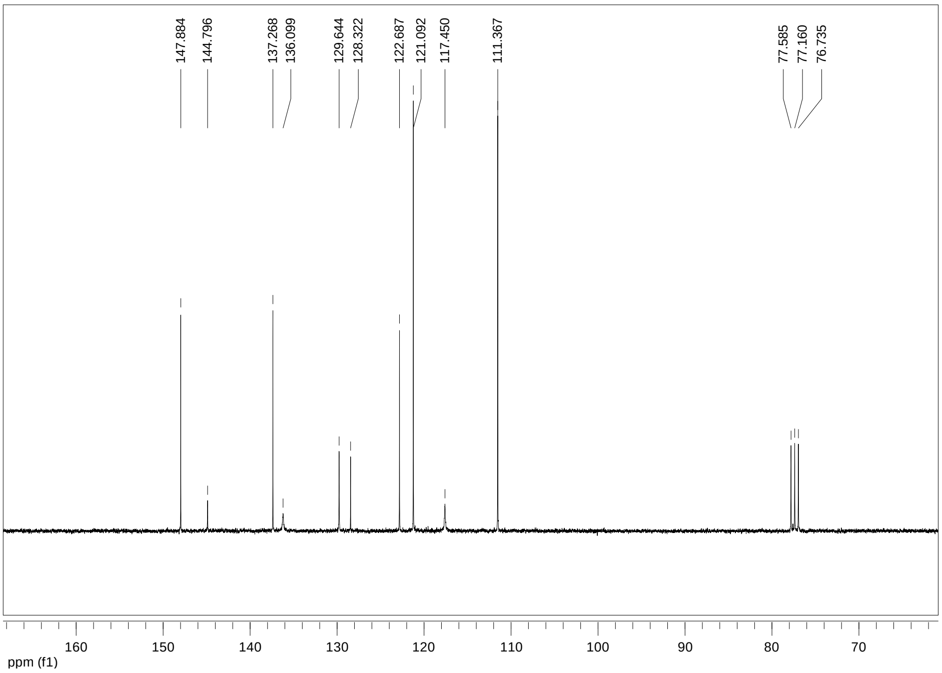

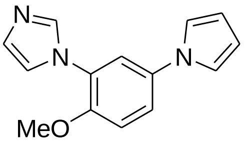

Compound 5d

^{1}H NMR (300 MHz, $CDCl_3$)

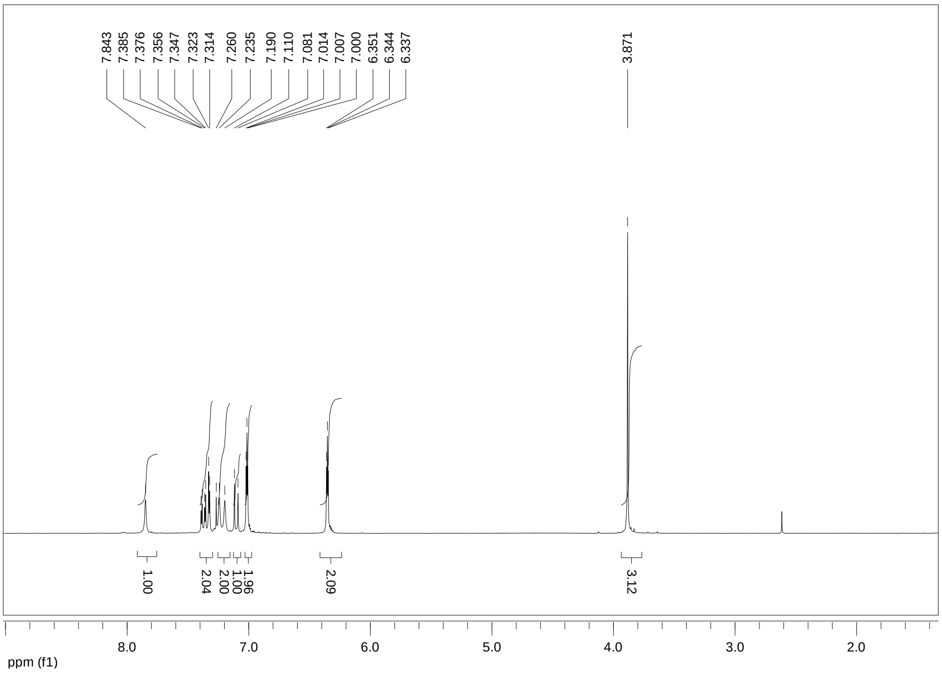

^{13}C NMR (75 MHz, $CDCl_3$)

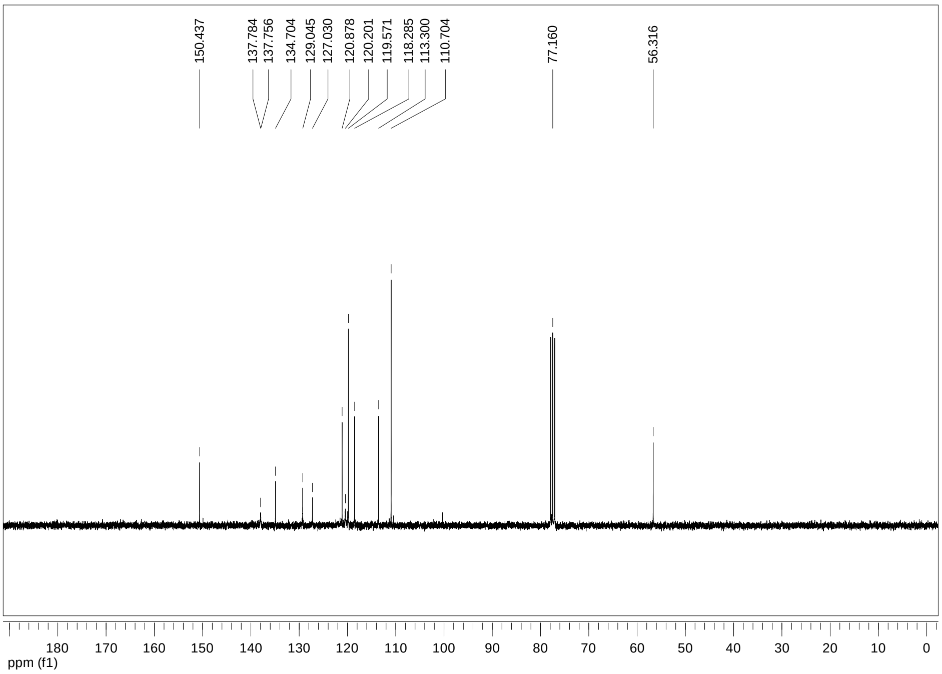

Compound 6d

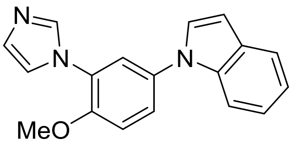

^{1}H NMR (300 MHz, $CDCl_3$)

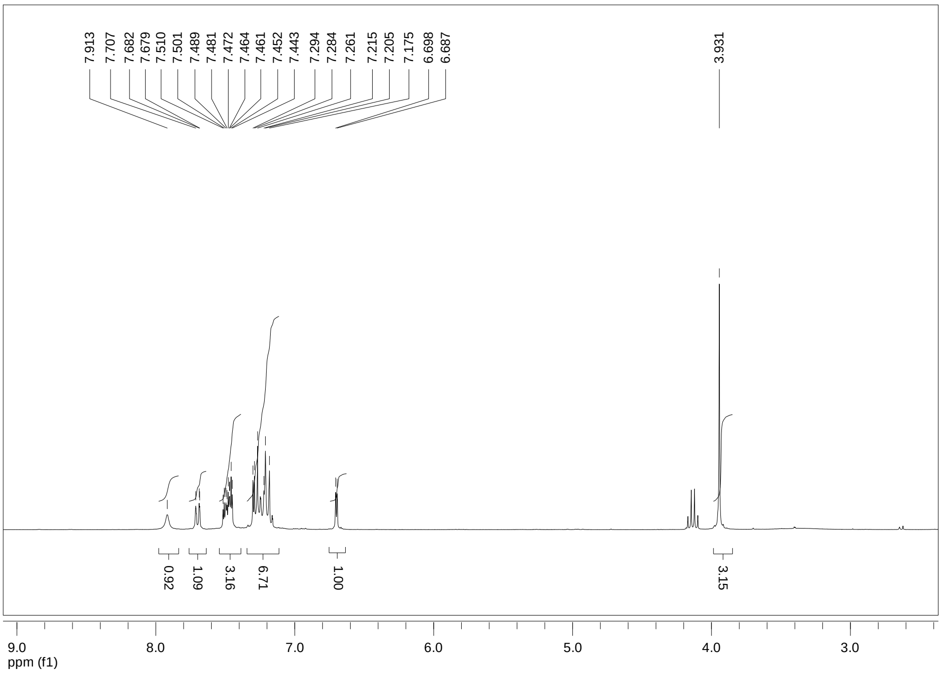

^{13}C NMR (75 MHz, $CDCl_3$)

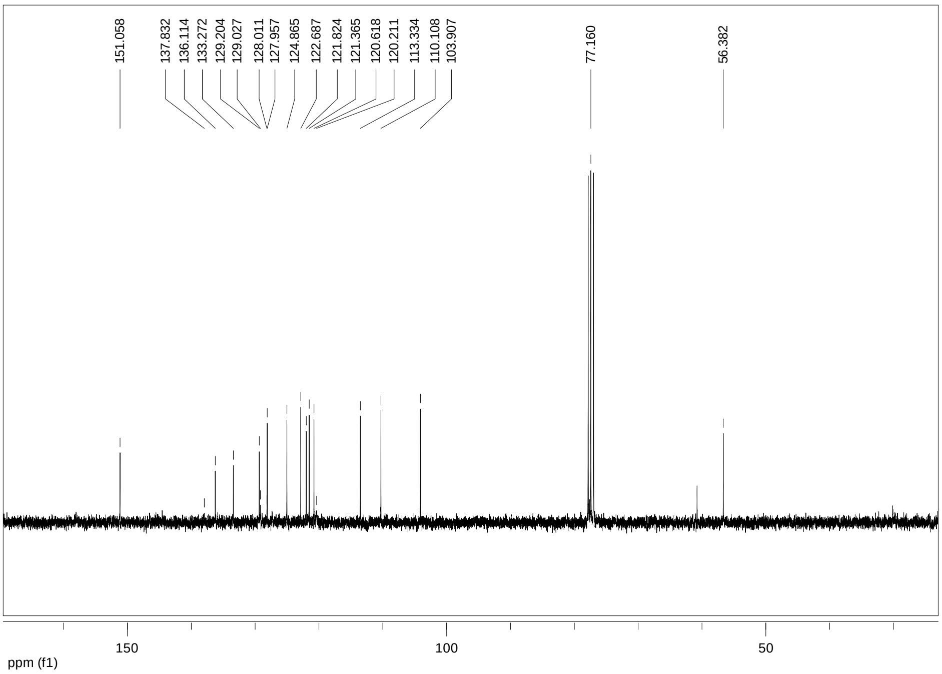

1a

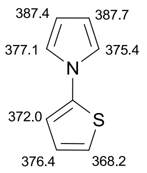

2a

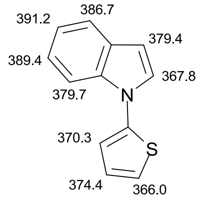

1b

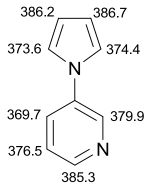

2b

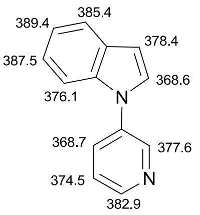

1c

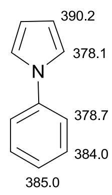

2c

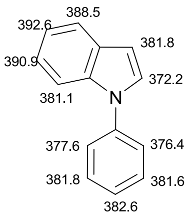

1d

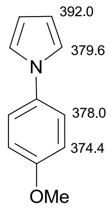

2d

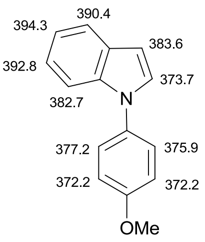

1e

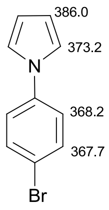

2e

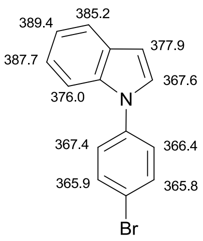

1f

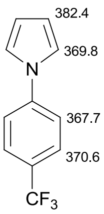

2f

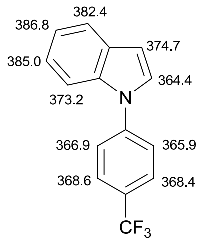

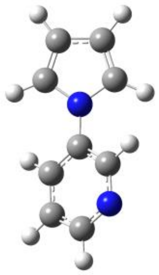

1b

C	-2.49745668	-0.10139721	0.84000820
C	-1.16969012	-0.22828081	0.51161267
C	-0.89897597	0.69698878	-0.54031589
C	-2.07250987	1.35155123	-0.82542282
N	-3.05801946	0.86670142	0.02090394
H	-3.10923691	-0.64480414	1.54463141
H	-0.47048172	-0.91638816	0.96717689
C	-4.40575778	1.28586004	0.04291307
C	-5.12316711	1.36368761	1.24107552
C	-5.06641676	1.63528461	-1.14479323
C	-6.45125886	1.77763551	1.19612548
H	-4.64141801	1.12389719	2.18391205
C	-7.00937595	2.11929394	-0.03645569
H	-7.03955760	1.85058216	2.10599111
H	-2.28881605	2.15212453	-1.51680592
H	0.05262872	0.86955601	-1.02458023
H	-4.54490663	1.55931099	-2.09672128
H	-8.04157986	2.45713411	-0.10357005
N	-6.33242017	2.05402791	-1.18953785

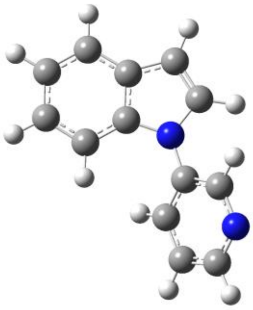

2b

C	0.32181200	2.00372700	0.30814300
C	1.68155500	2.12109600	0.32064600
C	2.22376400	0.80561800	0.11944200
C	1.12230700	-0.08471100	-0.00631800
H	-0.44541500	2.75054300	0.45519600
H	2.23727700	3.03715900	0.46712000
C	-1.37742300	0.20680300	0.05996700
C	-1.78926800	-0.93618700	0.75432500
C	-2.33729700	0.90501300	-0.68889300
C	-3.12207200	-1.32713500	0.65935600
H	-1.08272900	-1.48831700	1.36541800
C	-3.99874900	-0.55438300	-0.10367300
H	-3.47963900	-2.20833200	1.18371900
H	-2.04235800	1.78855400	-1.25239200
C	1.29954500	-1.44921200	-0.26224000
C	3.52917600	0.29623400	0.01811700
C	3.70872900	-1.06092100	-0.21481900
C	2.60368500	-1.92265800	-0.36020900
H	4.38445700	0.96013700	0.11399800
H	4.71383000	-1.46580600	-0.29461400
H	2.77097200	-2.97803100	-0.55688000
H	0.45473800	-2.11738100	-0.39402000
N	-0.04493000	0.67176600	0.11848300
H	-5.04788600	-0.82995300	-0.19084000
N	-3.62047400	0.54705200	-0.76416500
